# Targeting tumor-associated CCR2^+^ macrophages to inhibit pancreatic cancer recurrence following irreversible electroporation

**DOI:** 10.1126/sciadv.adw2937

**Published:** 2025-07-23

**Authors:** Weichen Xu, Shaoyue Li, Xuexia Shan, Qiao Wang, Xinhua Chen, Shengbo Wu, Yincheng Gao, Dandan Shan, Shisi Ding, Weiwei Ren, Xiaodong Hou, Shuo Liu, Taixia Wang, Yuting Shen, Zhiyuan Niu, Huixiong Xu, Liping Sun, Wenwen Yue

**Affiliations:** ^1^ Department of Medical Ultrasound, Center of Minimally Invasive Treatment for Tumor, Shanghai Tenth People’s Hospital, School of Medicine, Tongji University, Shanghai 200072, P. R. China.; ^2^ Key Laboratory of Pulsed Power Translational Medicine of Zhejiang Province, Hangzhou 311100, P. R. China.; ^3^ Department of Ultrasound, Zhejiang Hospital, Hangzhou, Zhejiang Province 310013, P. R. China.; ^4^ Department of Medical Ultrasound, Shanghai Hospital of Traditional Chinese Medicine, Shanghai 201899, P. R. China.; ^5^ Department of Ultrasound, Institute of Ultrasound in Medicine and Engineering, Zhongshan Hospital, Fudan University, Shanghai 200032, P. R. China.; ^6^ Department of Medical Ultrasound, Henan Provincial People’s Hospital, School of Medicine, Henan University, Zhengzhou, Henan 450003, P. R. China.

## Abstract

Pancreatic ductal adenocarcinoma (PDAC) is a highly lethal malignancy with pronounced resistance to conventional therapies. Irreversible electroporation (IRE) is a promising therapy for PDAC; however, its clinical efficacy is limited by a high recurrence rate. Here, using a preclinical PDAC model, we characterized the tumor immune microenvironment following insufficient IRE (iIRE) through single-cell RNA sequencing. We found that iIRE induces a CCR2^+^ tumor-associated macrophage (CCR2^+^ TAM)–mediated immunosuppressive microenvironment in residual tumors. Consequently, we developed a macrophage-based proteolipid vesicle (mPLV) coencapsulating the CCR2 antagonist PF-4136309 (PF) and gemcitabine (GEM), named PF/GEM@mPLV. Our findings suggest that PF/GEM@mPLV achieves high drug accumulation within tumors through iIRE-induced inflammation. Reduction of CCR2^+^ TAMs enhances antitumor immunity and improves chemotherapeutic response. PF/GEM@mPLV markedly inhibits tumor recurrence following iIRE, diminishes hepatic metastases, and prolongs survival in preclinical PDAC models. These findings uncover the role of CCR2^+^ TAMs in iIRE-induced immunosuppression, offering a promising strategy to enhance the clinical potential of IRE in PDAC.

## Teaser

Inhibition of a distinct macrophage subcluster is expected to hinder recurrence of pancreatic cancer after IRE ablation.

## INTRODUCTION

Pancreatic ductal adenocarcinoma (PDAC) typically presents with subtle early symptoms, and upon diagnosis, ~75 to 80% of patients are deemed unsuitable for surgical resection due to either local advancement or distant metastases, thereby establishing it as one of the most lethal human malignancies (*1*–*4*). The current established standard of care for advanced, unresectable PDAC involves chemoradiotherapy, stereotactic body radiation therapy, or chemotherapy alone; however, the efficacy remains limited (*5*–*7*). Currently, local ablation therapies such as radiofrequency ablation, microwave ablation, and irreversible electroporation (IRE) are increasingly used in the management of advanced PDAC, in accordance with international clinical guidelines (*7*, *8*). Among these techniques, IRE, which uses high-voltage electric fields to induce cell death through permanent membrane lysis, is a nonthermal ablation method. Compared with thermal ablation treatments, this approach has shown substantial efficacy in preserving critical anatomical structures such as major bile ducts and vessels, thereby substantially reducing the risk of complications including pancreatic fistula formation and hemorrhage (*9*). Consequently, IRE has emerged as a highly promising therapeutic modality for locally advanced pancreatic cancer. A multicenter study encompassing 200 patients with advanced PDAC showed a promising median overall survival of nearly 24 months with appropriate use of IRE treatment (*10*). Although IRE treatment offers certain advantages, the recurrence rates have ranged from 6 to 58% (*11*–*14*), posing a major challenge for the management of PDAC.

Because of the indistinct tumor margins and potential undetected micrometastases, IRE treatment, similar to other locoregional ablation therapies, often leaves behind a small fraction of residual tumor cells, which inevitably leads to recurrence (*15*–*17*). Accordingly, strategies to precisely uncover the underlying effects of IRE on the tumor microenvironment (TME) should be crucial to optimize the clinical response of IRE-based antitumor therapies for PDAC. Previously, our studies highlighted that inadequate thermal ablation induces intricate inflammatory responses within residual tumors, ultimately fostering the development of an immunosuppressive TME and facilitating tumor recurrence (*18*–*20*). Although the effect of thermal ablation on residual tumors has been demonstrated, the impact of IRE, a nonthermal ablation technique, on residual cancer cells remains largely unexplored. In particular, the development of single-cell sequencing [single-cell RNA sequencing (scRNA-seq)] technology enables us to characterize the tumor immune microenvironment and identify changes in the expression profiles of various immune cells (*21*–*23*).

In this study, using a preclinical murine model of PDAC, we characterized the tumor immune microenvironment following insufficient IRE (iIRE) treatment through scRNA-seq, flow cytometry, and multiple immunohistochemical analyses. Our results revealed that the CCR2^+^ subset of tumor-associated macrophages (TAMs) plays a critical role in establishing an immunosuppressive TME post-iIRE treatment, thereby facilitating rapid tumor recurrence. Previous studies have demonstrated that TAMs not only foster an immunosuppressive milieu that promotes tumor progression but also mediate resistance to chemotherapeutic agents, thereby posing a substantial obstacle to effective treatment (*21*, *24*–*27*). On the basis of these findings, we propose that therapeutics specifically targeting CCR2^+^ TAMs can be strategically used to modulate the TME of residual tumors following iIRE, with the objective of enhancing the therapeutic index of well-established treatments in PDAC.

**Fig. 1.**
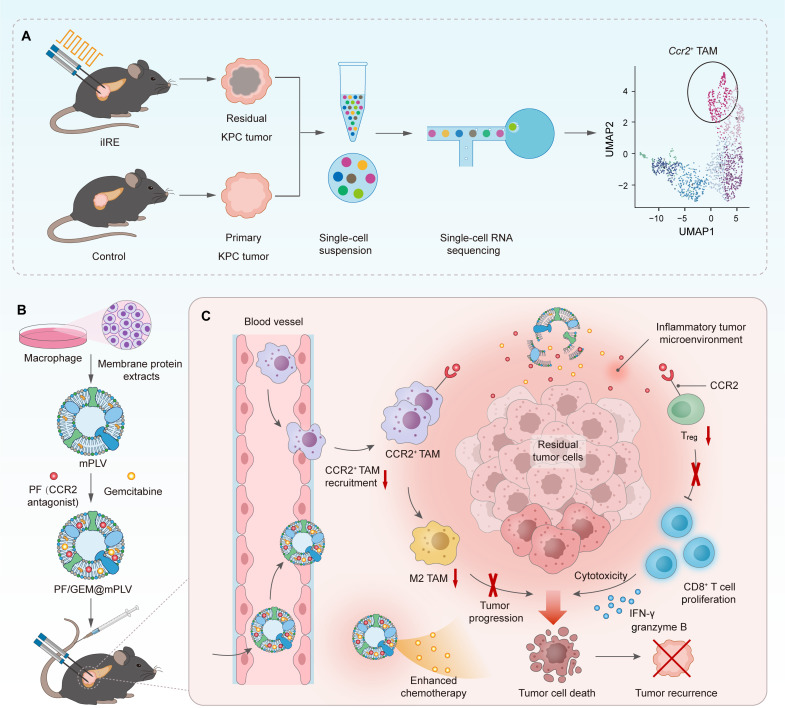
Design and mechanism of CCR2-targeted proteolipid nanovesicles for inhibiting pancreatic cancer recurrence following IRE. (**A**) Target identification using scRNA-seq. (**B**) Schematic illustration of the synthesis process of mPLVs loaded with CCR2 antagonist PF-4136309 (PF) and GEM, termed PF/GEM@mPLV. (**C**) Enhanced immune activation and cytotoxicity induced by PF/GEM@mPLV in combination with IRE therapy.

The efficacy of therapeutics for PDAC treatment is frequently constrained by the low bioavailability in tumor tissues, which results from the presence of a stromal barrier (*28*, *29*). Local tumor ablation induces direct apoptosis in PDAC cells and elicits an inflammatory response at the treatment site (*30*, *31*). In this context, we introduce inflammation-targeting nanovesicles (*32*) that leverage the inflammatory gradient generated following IRE treatment to facilitate drug accumulation within the tumor (*33*). Specifically, a macrophage-based proteolipid vesicle (mPLV) coloaded with the CCR2 antagonist PF-4136309 (PF) and the frontline chemotherapeutic agent gemcitabine (GEM) was developed. Macrophage membranes, owing to their inherent ability to target inflammatory tissues (*34*), have gained growing attention in the field of precision medicine. Considering the poor drug loading efficiency of macrophage membranes alone, liposomes composed of a phospholipid bilayer are used for fusion with the membrane. Our results demonstrated that the macrophage-based proteolipid vesicle loaded with PF and GEM (PF/GEM@mPLV) not only retain the excellent biocompatibility and inflammation-targeting capabilities of macrophage membranes but also effectively address the challenge of low drug loading efficiency. Following intravenous administration, the PF/GEM@mPLV proteolipid nanovesicles demonstrated targeted accumulation in tumors post-iIRE treatment. The combined treatment not only directly inhibited the recruitment of CCR2^+^ TAMs within the TME but also promoted the infiltration of CD8^+^ T cells while simultaneously reducing the accumulation of regulatory T cells (T_reg_ cells). Moreover, reducing the number of CCR2^+^ TAMs enhances the therapeutic efficacy of GEM. Our findings indicate that the combination of PF-induced CCR2^+^ TAM depletion and GEM treatment demonstrates a robust synergistic effect, markedly suppressing tumor recurrence after iIRE treatment and the formation of hepatic metastases, thereby significantly extending survival in mice with well-established orthotopic pancreatic tumors (Fig. 1). Given that all components of PF/GEM@mPLV are US Food and Drug Administration (FDA)–approved agents with the exception of the macrophage-derived membrane, our integrated treatment strategy exhibits substantial translational potential, particularly in addressing postablative tumor recurrence in patients with locally advanced PDAC.

## RESULTS

### Characterizing the immunosuppressive microenvironment of PDAC following iIRE treatment

In this study, we used a PDAC cell line derived from genetically engineered KPC (*Kras^G12D/+^*; *Trp53^R172H/+^*; *Pdx1-Cre*) mice (hereafter referred to as KPC cells) to establish preclinical PDAC models. To generate an effective IRE-induced recurrence model, we first evaluated the ablation efficacy. Using contrast-enhanced ultrasound (CEUS), a common clinical imaging modality, we visualized the extent of ablation between the sham surgery group and the ablation group (fig. S1A). In the sham surgery group, only a puncture tract was visible. In the ablation group, we observed the loss of perfusion in the central region immediately following ablation treatment, with only minimal residual perfusion at the periphery. The tumor center lost perfusion completely with minimal blood flow in the periphery. In addition, histological examination demonstrated relatively complete central necrosis with residual viable cells at the margins (fig. S1B). These findings indicate the establishment of an in vivo iIRE model that mimics clinical features of postablation tumor recurrence.

**Fig. 2.**
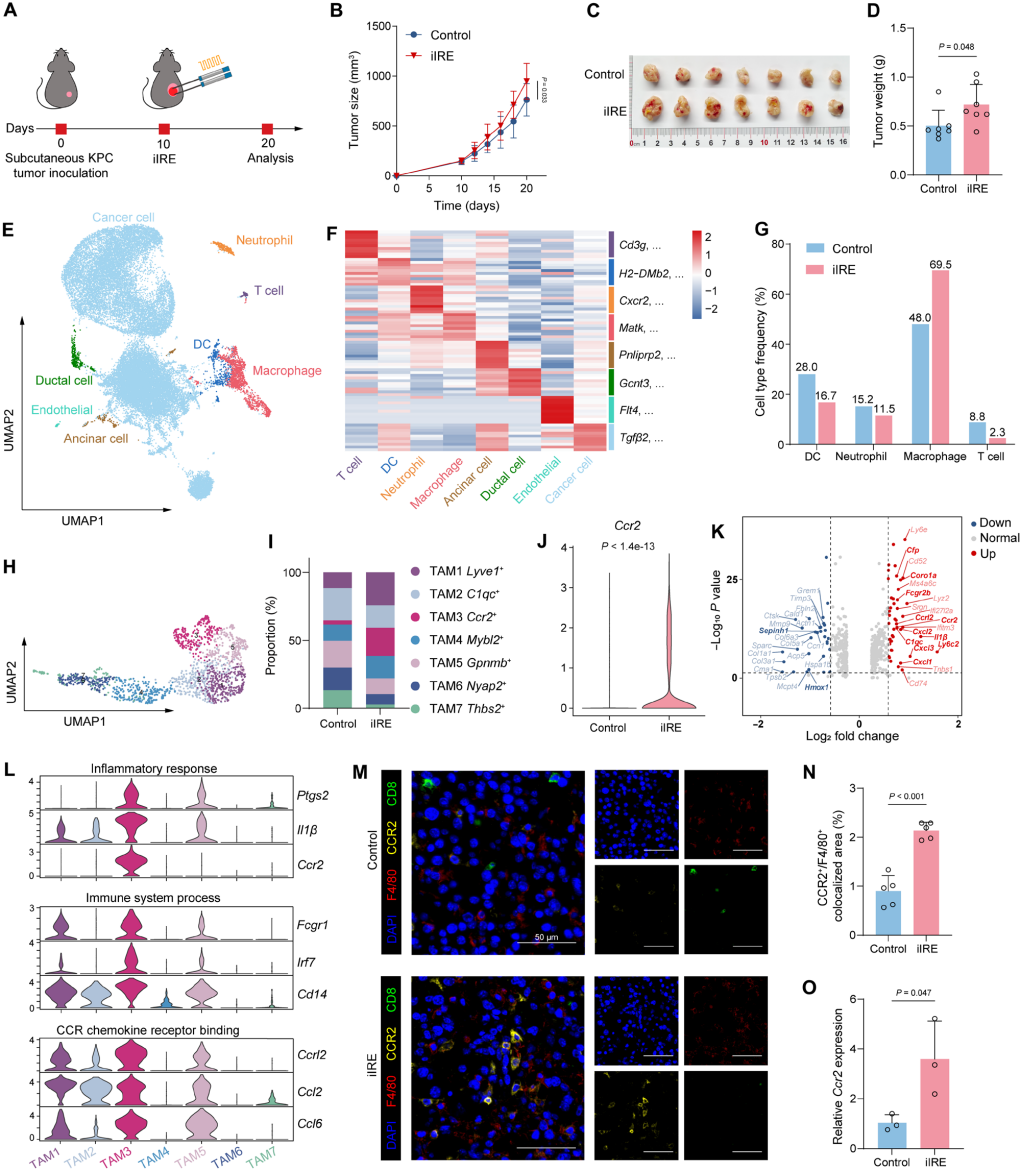
Characterization of the immunosuppressive microenvironment of PDAC following iIRE treatment. (**A**) Experimental timeline for the subcutaneous KPC tumor model. (**B**) Tumor growth curve (*n* = 7). (**C**) Gross images of tumors. (**D**) Tumor weights (*n* = 7). (**E**) Uniform Manifold Approximation and Projection (UMAP) plot of eight cell types. (**F**) Heatmap of selected marker genes for different cell types. (**G**) Percentage of immune cells. (**H**) Reclustering of TAMs displayed by UMAP plot. (**I**) Proportion of seven TAM subclusters. (**J**) Violin plot displaying *Ccr2* expression levels between the control and iIRE groups in TAMs. (**K**) Volcano plot showing the top 20 differentially expressed genes (DEGs) in TAMs between the control and iIRE groups; key genes are highlighted in bold; vertical dotted lines represent |log_2_ fold change| > 0.5. (**L**) Expression of selected genes belonging to the indicated categories in TAM subclusters. (**M**) Representative multiplex immunofluorescence (mIF) staining images of tumor sections (scale bars, 50 μm); 4′,6-diamidino-2-phenylindole (DAPI; blue), F4/80 (red), CCR2 (yellow), and CD8 (green). (**N**) Quantification of CCR2^+^F4/80^+^ colocalized pixels relative to the total area [*n* = 5 fields of view (FOVs)]. (**O**) Validation using quantitative polymerase chain reaction (qPCR) of *Ccr2* expression in KPC tumors treated with or without iIRE (*n* = 3). Data are expressed as mean ± SD. Statistical differences were calculated using Student’s *t* test.

To investigate the impact of iIRE on tumor progression, we used a subcutaneous KPC PDAC mouse model in this study. When the tumor length reached 7 to 9 mm, iIRE ablation was performed to partially remove the tumor (Fig. 2A). Afterward, the tumor burden was monitored by caliper measurement. Our findings indicated that the post-iIRE residual tumor exhibited a more rapid growth rate compared to the untreated control group (Fig. 2, B and C). As depicted in Fig. 2D, the changes in tumor weight on day 10 post-iIRE were consistent with tumor volume. These results together suggest that the iIRE-induced tissue injury can facilitate the rapid proliferation of residual tumors.

To further mimic the pathogenesis of pancreatic cancer, we established an orthotopic KPC tumor model for the follow-up experiments (fig. S2, A and B). Similarly, iIRE ablation was performed on the orthotopic tumors (fig. S2C). To objectively understand the underlying molecular mechanisms of the potential prooncogenic effect of iIRE, we performed scRNA-seq on residual tumors isolated 3 days post-iIRE and on untreated tumors as controls. After quality control and screening, we obtained a total number of 16,031 cells from the control group and 16,944 from the iIRE group. The average number of unique molecular identifiers (UMIs) per cell was 10,452 in the control group and 12,903 in the iIRE group, while the average gene counts per cell were 3068 and 3410, respectively. According to the known lineage marker genes (ImmGen dataset), we identified malignant cancer cells and seven types of nonmalignant cells, including T cells, dendritic cells (DCs), neutrophils, macrophages, acinar cells, ductal cells, and endothelial cells (Fig. 2E and fig. S3). The gene expressions of the top five markers for these cell types are shown in Fig. 2F. An increase in macrophages was observed among the four immune cell types in the iIRE group compared to the control group, while a decrease was noted in lymphocytes (particularly CD8^+^ T cells) (Fig. 2G, Table 1, and figs. S4 and S5). We further validated the changes in macrophage and neutrophil proportion using flow cytometry. The results confirmed a significant increase in macrophages and neutrophils post-iIRE (fig. S6), consistent with previous studies (*16*, *33*). In addition, CellChat analysis (*35*) was conducted to investigate ligand-receptor interactions among different cell subpopulations. Compared to the control group, the iIRE group exhibited increased cell-cell interaction strengths among immune cell populations (fig. S7, A and B). Notably, interactions between macrophages and T cells, as well as neutrophils, were more active in the iIRE group. These interactions involved chemokine-related C-C motif chemokine ligand (CCL) and CXCL pathways and pro-inflammatory migration inhibition factor pathways (fig. S7, C to E). The increased activity of these specific cellular interactions underscores the pro-inflammatory alterations induced by iIRE and highlights the critical role of macrophages in regulating the TME after iIRE treatment.

**Table 1.** Cell number and frequency.

Sample ID	New cell type	Cell number	Frequency
Control	T cell	95	0.592602
Control	DC	303	1.890088
Control	Neutrophil	164	1.023018
Control	Macrophage	519	3.237477
Control	Ancinar cell	240	1.497099
Control	Ductal cell	176	1.097873
Control	Endothelial	35	0.218327
Control	Cancer cell	14,499	90.44352
iIRE	T cell	35	0.206563
iIRE	DC	249	1.469547
iIRE	Neutrophil	172	1.015109
iIRE	Macrophage	1,038	6.126062
iIRE	Ancinar cell	85	0.501653
iIRE	Ductal cell	170	1.003305
iIRE	Endothelial	31	0.182956
iIRE	Cancer cell	15,164	89.49481

### CCR2^+^ TAM as a therapeutic target against post-iIRE tumor recurrence

Given the substantial accumulation of macrophages in residual tumors post-iIRE, we conducted a reclustering analysis of the macrophage population within PDAC tumors to identify distinct subsets of TAMs (Fig. 2H). On the basis of the expression of their marker genes, a total of seven distinct TAM subclusters were identified: *Lyve1^+^* TAMs express genes consistent with tissue-resident macrophages (*Mrc1*, *Selenop*, and *Siglec1*) (*36*), *C1qc^+^* TAMs express genes related to inflammatory responses (*Aif1* and *C1qa*), *Ccr2^+^* TAMs express genes associated with macrophage recruitment and activation (*Ly6c2* and *Trem1*), *Mybl2^+^* TAMs express cell-cycle–related genes (*Mki67* and *Plcaf*), *Gpnmb^+^* TAMs express genes related to lipid metabolism (*Spp1* and *Cd36*), *Nyap2^+^* TAMs express genes involved in signal transduction, and *Thbs2^+^* TAMs express proteins related to extracellular matrix (*Sepinh1*) (fig. S8A). Notably, the proportion and number of *Ccr2*^+^ TAMs evidently increased in the iIRE group compared to the control group (Fig. 2I and fig. S8, B and C). We subsequently screened differentially expressed genes (DEGs) in TAMs between the two groups. The *Ccr2* gene was significantly up-regulated in the iIRE group (Fig. 2J). Moreover, other important inflammation-related genes such as *Cfp*, *C1qc*, *Coro1a*, *Il1*β, and *Cxcl2* were also up-regulated, while the anti-inflammatory gene *Hmox1* that helps maintain immune homeostasis was down-regulated (Fig. 2K).

CCR2 is a well-established surface marker of bone marrow–derived monocytes or macrophages (*37*). In the TME, elevated CCL2-mediated chemotaxis recruits CCR2^+^ circulating monocytes to the tumor site, thereby contributing to the establishment of an immunosuppressive TME (*38*, *39*). To investigate the distinct characteristics of CCR2^+^ TAMs, we conducted a Gene Ontology (GO) analysis. The results revealed that *Ccr2*^+^ TAMs were enriched in pathways including inflammatory response (*Ptgs2*, *Il1*β, and *Ccr2*), immune system processes (*Fcgr1*, *Irf7*, and *Cd14*), and CCR chemokine receptor binding (*Ccrl2*, *Ccl2*, and *Ccl6*) (Fig. 2L). To further elucidate the downstream signaling pathways, we conducted gene set enrichment analysis (GSEA) on the DEGs. Our results showed that the nuclear factor κB signaling, tumor necrosis factor (TNF) signaling, Nod-like receptor signaling, chemokine signaling, and Toll-like receptor pathways were significantly enriched in the iIRE group (fig. S9). Besides, the multiplex immunofluorescence (mIF) staining analysis further confirmed the high infiltration of CCR2^+^ TAMs (CCR2^+^F4/80^+^) and a reduction in CD8^+^ T cells (Fig. 2, M and N) in the residual post-iIRE tumor. We subsequently validated *Ccr2* expression changes by quantitative polymerase chain reaction (qPCR), which showed a 3.5-fold up-regulation in the iIRE group compared to the control group (Fig. 2O).

**Fig. 3.**
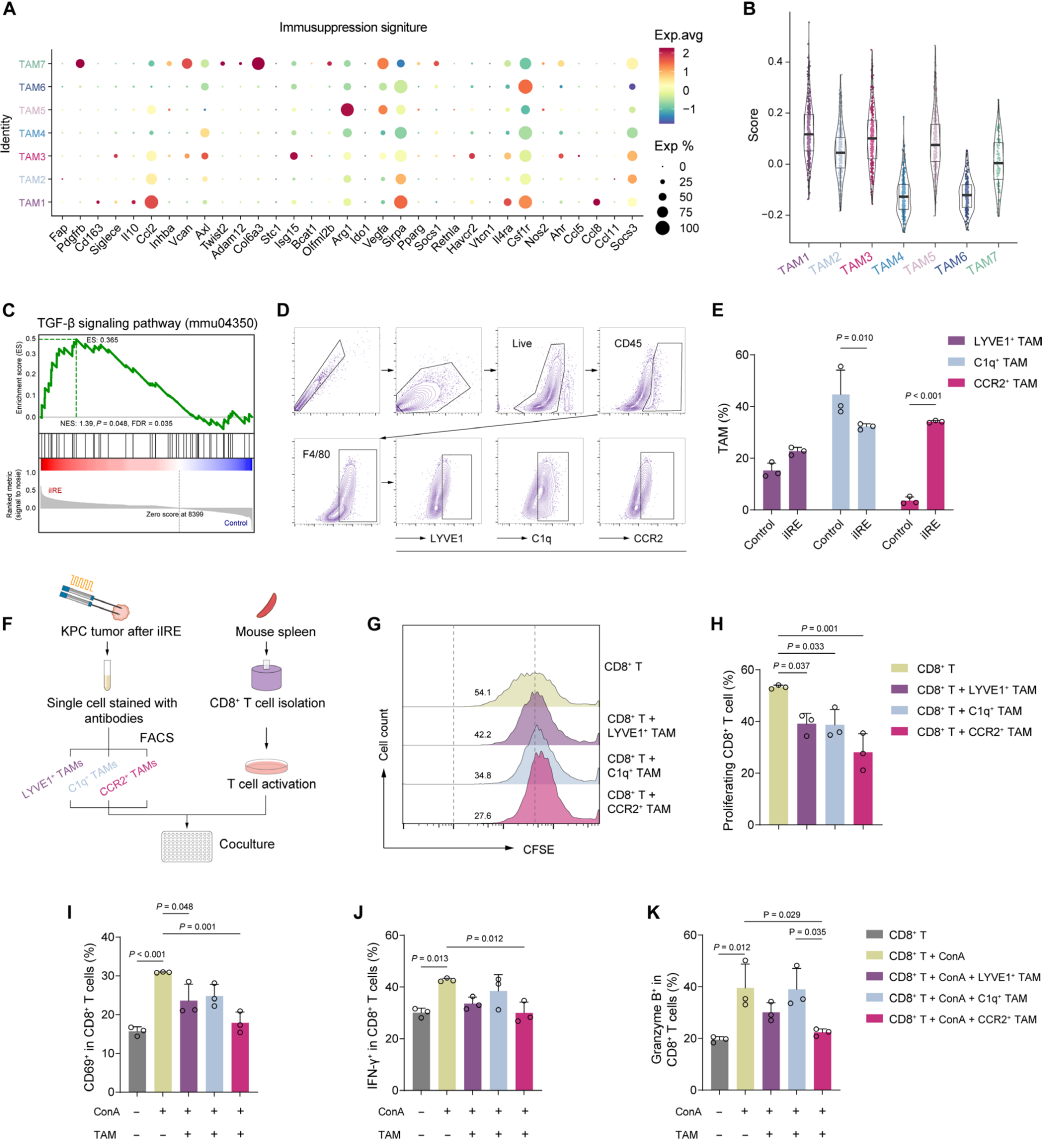
Validation of the immunosuppressive potential of CCR2^+^ TAM. (**A**) Bubble plot showing the expression of immunosuppressive genes across seven TAM subclusters. (**B**) Immunosuppressive scores. (**C**) GSEA of the TGF-β signaling pathway in *Ccr2*^+^ TAMs between the control and iIRE groups. FDR, false discovery rate. NES, normalized enrichment score. (**D**) Gating strategy for flow cytometry and FACS of LYVE1^+^ TAMs, C1q^+^ TAMs, and CCR2^+^ TAMs. (**E**) Quantitative analysis of TAM subcluster proportions by flow cytometry (*n* = 3). (**F**) Schematic illustration of the CD8^+^ T cell suppression assay. (**G**) Representative histograms of carboxyfluorescein diacetate succinimidyl ester (CFSE)–labeled T cells. (**H**) Quantification of proliferating CD8^+^ T cells (*n* = 3). (**I** to **K**) Flow cytometric analysis of CD69^+^ (I), IFN-γ^+^ (J), and granzyme B^+^ (K) CD8^+^ T cells (*n* = 3). Data are expressed as mean ± SD. Statistical differences were calculated using two-way analysis of variance [ANOVA (E)] and one-way ANOVA (H to K).

To assess the immunosuppressive potential of CCR2^+^ TAMs relative to other TAM subclusters, we curated a panel of more than 30 genes related to immune suppression (*40*–*42*), including *Fap*, *Siglece*, *Il10*, *Isg15*, *Arg1*, *Ido1*, etc., and assessed the expression of this gene set across different TAMs (Fig. 3A). By quantifying the immunosuppression score applying AddModuleScore analysis, the results showed that TAM1 (*Lyve1^+^*), TAM2 (*C1qc^+^*), TAM3 (*Ccr2*^*+*)^, and TAM5 (*Gpnmb^+^*) exhibited relatively high scores (Fig. 3B). In *Ccr2*^+^ TAM, the transforming growth factor–β (TGF-β) signaling pathway—associated with immune suppression—was significantly enriched in the iIRE group (Fig. 3C). Before cell sorting, we validated the proportional changes in these TAM subclusters after iIRE treatment (Fig. 3D). However, *Gpnmb*^+^ TAMs (TAM5) were not included because flow cytometry antibody was not available. Proportional changes after iIRE revealed increased CCR2^+^ and lymphatic vessel endothelial hyaluronan receptor 1 (LYVE1)^+^ TAMs and decreased complement component 1q (C1q)^+^ TAMs (Fig. 3E), consistent with scRNA-seq data. Next, to functionally validate the immunosuppressive potential of these TAM subclusters, we isolated TAMs from tumor tissue after iIRE treatment via fluorescence-activated cell sorting (FACS) and CD8^+^ T cells from mouse spleens using magnetic-activated cell separation for a coculture suppression assay (Fig. 3F). In coculture assays (CD8^+^ T: TAM = 10:1) with CD3/CD28 antibody stimulation, all three TAMs suppressed CD8^+^ T cell proliferation to varying degrees, with CCR2^+^ TAMs showing a stronger inhibitory effect (Fig. 3, G and H). In cytotoxicity validation with CD3/CD28 and concanavalin A (ConA) stimulation, CCR2^+^ and LYVE1^+^ TAMs significantly reduced CD69 expression on CD8^+^ T cells, while only CCR2^+^ TAMs markedly suppressed interferon-γ (IFN-γ) and granzyme B production (Fig. 3, I to K, and fig. S10). Collectively, these results demonstrate that immunosuppressive capacity is not exclusive to CCR2^+^ TAMs, but CCR2^+^ TAMs exert the most potent inhibitory effect on CD8^+^ T cell function, underscoring their relevance as a key immunotherapeutic target. Therefore, therapeutics that actively target CCR2^+^ TAMs may be strategically used to optimize the subversion of tumor immunosuppression, thereby effectively combating post-iIRE tumor recurrence.

### Preparation and characterization of PF/GEM@mPLV

To inhibit the recruitment of CCR2^+^ TAMs to the tumor site following iIRE, we incorporated a highly selective CCR2 inhibitor (PF-4136309), which is currently in phase 2 clinical trials for advanced PDAC (*43*), into our therapeutic strategy. Furthermore, GEM is a commonly used first-line chemotherapeutic agent for PDAC. However, previous studies have shown that TAMs contribute to the resistance of tumor cells to GEM through mechanisms such as pyrimidine secretion and exosome release (*24*–*26*). Therefore, using PF to inhibit CCR2^+^ TAM–induced immunosuppression is expected to enhance the therapeutic index of GEM and holds promise for mitigating post-iIRE tumor recurrence. To enhance the concentration of free drugs in tumors, we developed an mPLV by incorporating macrophage membrane proteins into phospholipid bilayers. This mPLV coencapsulates PF and GEM, resulting in the formation of PF/GEM@mPLV. We hypothesize that the inflammatory gradient generated by iIRE treatment promotes the migration and accumulation of PF/GEM@mPLV at the tumor site.

**Fig. 4.**
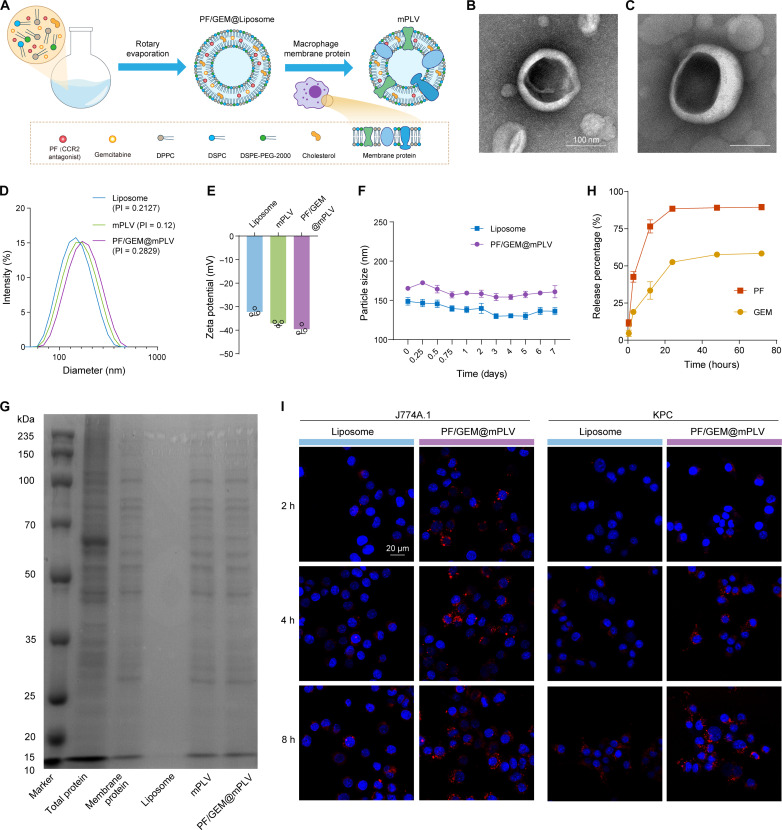
Synthesis and characteristics of PF/GEM@mPLVs. (**A**) Schematic diagram of the PF/GEM@mPLV synthesis process. (**B** and **C**) Representative TEM images of (B) liposome and (C) mPLV (scale bar, 100 nm). PI, polydispersity index. (**D**) Representative particle-size distributions of liposome, mPLV, and PF/GEM@mPLV. (**E**) Zeta potentials of liposome, mPLV, and PF/GEM@mPLV (*n* = 3). (**F**) Particle size variations over 7 days of liposome and PF/GEM@mPLV (*n* = 3). (**G**) SDS–polyacrylamide gel electrophoresis (SDS-PAGE) analysis of the protein composition of macrophage total proteins, macrophage membranes, liposomes, mPLVs, and PF/GEM@mPLVs, respectively. (**H**) Drug release curve of PF and GEM (*n* = 3). (**I**) Representative images of 1,1′-dioctadecyl-3,3,3′,3′-tetramethylindocarbocyanine perchlorate (DiI)–labeled particles internalized by macrophages (J774A.1 cell line) and KPC cells at different time points by confocal laser scanning microscope (CLSM; scale bar, 20 μm). h, hours. Data are expressed as mean ± SD.

As illustrated in Fig. 4A, the proteolipid nanovesicles were prepared using thin-film evaporation, followed by extrusion. The mass ratio of membrane proteins to lipids was 1:300. Transmission electron microscopy (TEM) revealed that liposomes, mPLVs, PF/GEM@Liposomes, and PF/GEM@mPLVs all exhibit bilayer, spherical lipid structures (Fig. 4, B and C, and fig. S11). Dynamic light scattering (DLS) measurements showed an average liposome particle size of 148.8 ± 4.1 nm. The average sizes of mPLVs and PF/GEM@mPLVs were slightly larger, measuring 170.0 ± 2.1 and 172.2 ± 1.9 nm, respectively (Fig. 4D). The average ζ potentials of liposomes, mPLVs, and PF/GEM@mPLVs in hydrated solution were −32.3 ± 1.2, −37.1 ± 0.8, and −39.6 ± 1.5 mV, respectively (Fig. 4E). The polydispersity index for all three particles was ~0.2, indicating their good dispersibility. Notably, over a period of 7 days, no significant changes in particle size were observed in phosphate-buffered saline (PBS) solution at 37°C (pH 7.4), thereby demonstrating their good in vitro stability (Fig. 4F).

SDS–polyacrylamide gel electrophoresis (SDS-PAGE) demonstrated that the protein profiles of PF/GEM@mPLV were consistent with those of macrophage membrane proteins, thereby validating the successful binding of macrophage membrane proteins to the surface of liposomes (Fig. 4G). The ultraviolet-visible (UV-vis) absorption spectrum of PF/GEM@mPLV exhibited a characteristic absorption peak at 269 nm, confirming the successful encapsulation of GEM into nanovesicles (fig. S12A). The successful coloading of PF was confirmed by high-performance liquid chromatography (HPLC) at 245 nm (fig. S12, B and C). The average encapsulation efficiencies (EE%) and drug loading capacities (DLC%) of PF and GEM in PF/GEM@mPLV were 75.4% and 6.3 wt % and 34.8% and 2.9 wt %, respectively (fig. S13). Drug release was monitored by dialysis at 37°C (pH 7.4), demonstrating a sustained release of both drugs into the medium (Fig. 4H). Overall, the physical properties of PF/GEM@mPLV, including nanoscale size and high dispersity, enhance subsequent in vitro performance evaluation and biomedical applications in vivo.

### In vitro evaluation of the PF/GEM@mPLV-mediated reduction in macrophage recruitment

Next, we aimed to assess the PF/GEM@mPLV-mediated reduction in macrophage recruitment in vitro. Initially, the interaction between PF/GEM@mPLV and KPC cells, as well as J774A.1 macrophages, was investigated. 1,1′-dioctadecyl-3,3,3′,3′-tetramethylindocarbocyanine perchlorate (DiI)–labeled liposomes or PF/GEM@mPLVs were cocultured with KPC cells and J774A.1 macrophages for 2, 4, and 8 hours, respectively. As expected, PF/GEM@mPLVs demonstrated a markedly enhanced internalization compared to conventional liposomes (Fig. 4I). In addition, as indicated in fig. S14, the cytotoxicity of the mPLV nanocarrier remained low at lipid concentrations up to 200 μg/ml and membrane protein concentrations up to 100 μg/ml, indicating its excellent biocompatibility.

**Fig. 5.**
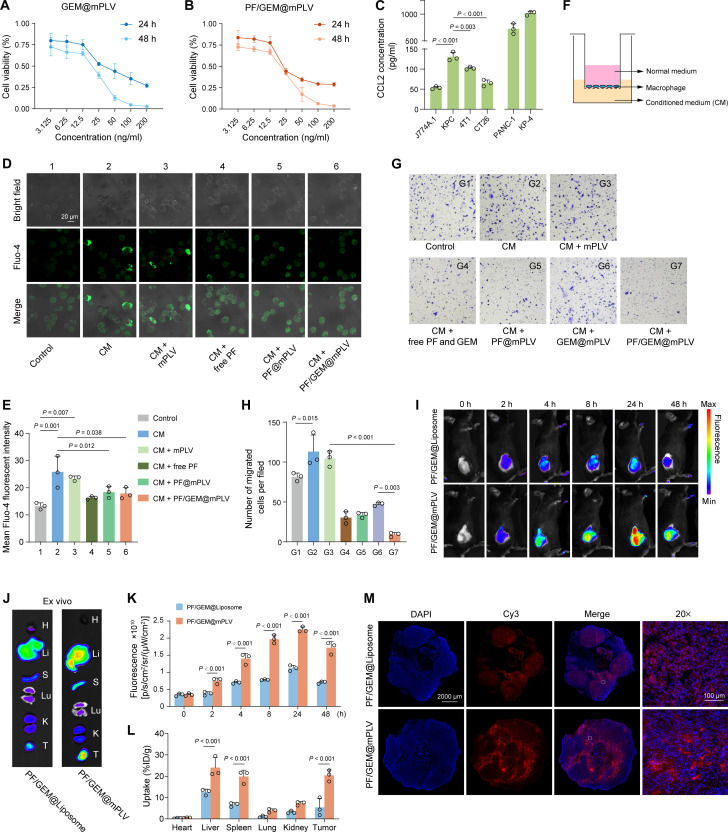
In vitro assays of PF/GEM@mPLVs and validation of targeting capability. (**A** and **B**) Cell viability of KPC cells treated with varying concentrations of GEM in (A) GEM@mPLVs or (B) PF/GEM@mPLVs. (**C**) Bar plots displaying CCL2 concentrations in the supernatants of mouse cell lines (J774A.1, KPC, 4T1, and CT26) and human cell lines (PANC-1 and KP-4) (*n* = 3). (**D**) Representative fluorescence images of J774A.1 cells stained with Fluo-4 AM. (**E**) Quantitative analysis of mean Fluo-4 fluorescence intensity (*n* = 3). (**F**) Schematic diagram of in vitro macrophage migration assay using a transwell system. (**G** and **H**) Representative images of migrated cells stained by crystal violet and quantification. (**I**) In vivo fluorescence images of KPC tumor–bearing mice captured at 0, 2, 4, 8, 24, and 48 hours after intravenous administration of Cy5.5-labeled PF/GEM@Liposome or PF/GEM@mPLV. (**J**) Representative ex vivo fluorescence images of major organs and tumors (H represents heart, Li represents liver, Lu represents lung, K represents kidney, T represents tumor, and S represents spleen) at 48 hours after administration of Cy5.5-labeled PF/GEM@Liposome or PF/GEM@mPLV. (**K**) Quantification of in vivo fluorescence (*n* = 3). (**L**) Quantification of nanoparticle biodistribution in major organs and tumor tissues, expressed as %ID/g (*n* = 3). (**M**) Representative fluorescence images of intratumoral permeation of Cy3-labeled nanoparticles in the whole tumor tissues 24 hours after injection. DAPI (blue) and Cy3 (red). Scale bars, 2000 μm (0.6× images) and 100 μm (20× images). Data are expressed as mean ± SD. Statistical differences were calculated using one-way ANOVA (C, E, and H) and two-way ANOVA (K and L).

To validate the direct cytotoxic effects of PF/GEM@mPLV on tumor cells, we performed Cell Counting Kit-8 (CCK-8) assays to assess cell viability. After 24 and 48 hours of different treatments on KPC cells, the median inhibitory concentration (IC_50_) values of GEM@mPLV were 39.26 and 13.77 ng/ml, respectively, while the IC_50_ values of PF/GEM@mPLV were 31.90 and 15.41 ng/ml (Fig. 5, A and B). The cytotoxicity of both GEM@mPLV and PF/GEM@mPLV showed a time- and GEM-dose–dependent enhancement.

Before validating the chemotaxis inhibitory effects of PF/GEM@mPLV, the concentration of the CCR2 ligand, CCL2, was quantified in the supernatants of multiple cell lines using enzyme-linked immunosorbent assay (ELISA). Our results showed that the CCL2 concentration in the KPC cell supernatant was significantly higher compared to that of J774A.1 macrophages, 4T1 breast cancer cells, and CT26 colon cancer cells (Fig. 5C). Similarly, a relatively high level of CCL2 expression was also observed in two human pancreatic cancer cell lines, PANC-1 and KP-4. We further explored the in vitro effects of PF/GEM@mPLV on macrophage recruitment. First, we derived conditioned medium (CM) from KPC cells as a stimulant or chemoattractant rich in CCL2 and other cytokines. For calcium influx assays, J774A.1 cells were incubated with different treatments for 1 hour. CM alone significantly increased intracellular calcium. Free PF, PF@mPLV, and PF/GEM@mPLV all significantly suppressed CM-induced calcium influx, indicating effective CCR2 signaling inhibition (Fig. 5, D and E). For the transwell assay, CM was added to the lower chamber, while macrophages were seeded in the upper chamber using serum-free medium to establish a chemotactic gradient (Fig. 5F). Subsequently, the cells were treated with various drugs. The results showed a significant increase in the number of migrated macrophages when cocultured with CM from KPC cells, as compared to macrophages cultured alone. Treatment with the mPLV did not significantly affect the number of migrated cells. However, free drugs (PF + GEM), PF@mPLV, GEM@mPLV, and PF/GEM@mPLV significantly reduced the number of migrated cells, with PF/GEM@mPLV showing the most pronounced reduction (Fig. 5, G and H). These findings confirmed that the combination therapy of PF/GEM@mPLV effectively inhibits CCL2-induced chemotaxis in J774A.1 cells.

### PF/GEM@mPLV inhibits post-iIRE tumor recurrence

Encouraged by the in vitro results, the efficacy of PF/GEM@mPLV to prevent tumor recurrence after iIRE was evaluated. Before the therapeutic experiment, the in vivo accumulation of Cy5.5-labeled PF/GEM@mPLV in post-iIRE tumor tissue was investigated using an in vivo imaging system (IVIS). The results demonstrated that a significant accumulation of PF/GEM@mPLV was observed in the tumor tissue, reaching its peak at 24 hours and subsequently declining (Fig. 5, I and K). The distribution of the nanovesicles in major organs and tumor tissues was evaluated 48 hours after administration. The highest concentrations were detected in the liver and tumor tissues, followed by the spleen, kidneys, and lungs, with the lowest concentration in the heart (Fig. 5, J and L). Compared to conventional liposomes, the PF/GEM@mPLV demonstrated significantly enhanced penetration and accumulation in the residual post-IRE tumor (Fig. 5M), attributed to its superior ability to target inflamed tissues.

**Fig. 6.**
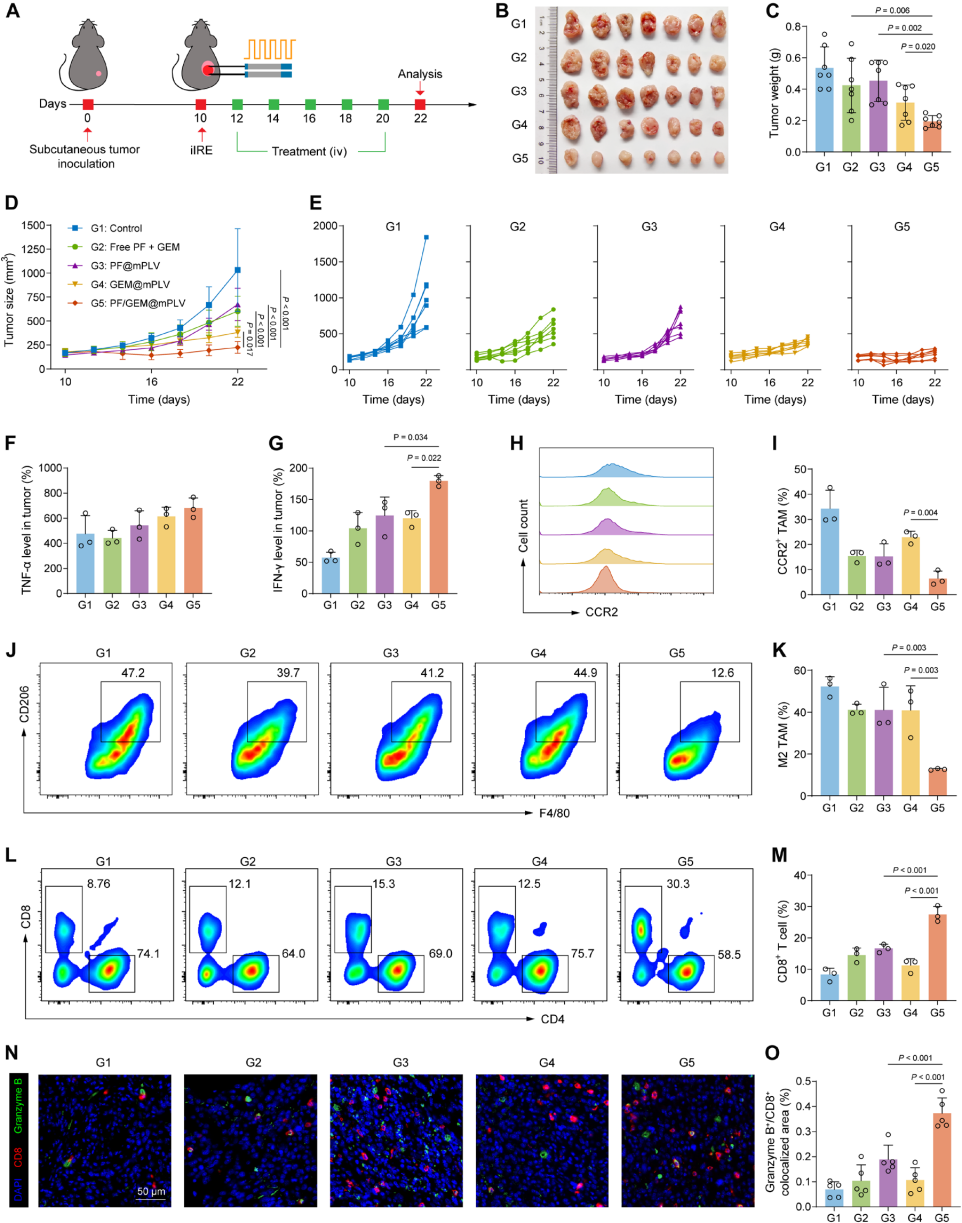
PF/GEM@mPLVs suppress tumor recurrence in subcutaneous KPC tumors and modulate immune response. (**A**) Schematic diagram of treatment strategy in subcutaneous KPC tumors. iv, intravenous. (**B**) Gross images of tumors in control (G1), free PF + GEM (G2), PF@mPLV (G3), GEM@mPLV (G4), and PF/GEM@mPLV (G5) groups. (**C**) Tumor weights in different treatment groups (*n* = 7). (**D** and **E**) Tumor growth curves (*n* = 7), from days 10 to 22. (**F** and **G**) Concentration of TNF-α and IFN-γ in tumor homogenates (*n* = 3). (**H** and **I**) Representative flow cytometric histogram of CCR2^+^ TAM (CCR2^+^Ly6G^−^F4/80^+^CD11b^+^CD45^+^) and quantification (*n* = 3). (**J** and **K**) Representative flow cytometric analysis of M2-TAM (CD206^hi^Ly6G^−^F4/80^+^CD11b^+^CD45^+^) and quantification (*n* = 3). (**L** and **M**) Representative flow cytometric analysis of CD8^+^ T cell (CD8^+^CD3^+^CD45^+^) and quantification (*n* = 3). (**N**) Representative mIF images of tumor tissues showing DAPI (blue), CD8 (red), and granzyme B (green) (scale bar, 50 μm). (**O**) Quantification of granzyme B^+^CD8^+^ colocalized pixels relative to the total area (*n* = 5 FOVs). Data are expressed as mean ± SD. Statistical differences were calculated using one-way ANOVA.

We further assessed the in vivo antitumor efficacy of PF/GEM@mPLV. As outlined in Fig. 6A, on day 10 postsubcutaneous inoculation with KPC tumors, the visible tumor was subjected to partial treatment with iIRE. Then, the tumor-bearing mice were randomly divided into five groups: control (G1), free PF + GEM (G2), PF@mPLV (G3), GEM@mPLV (G4), and PF/GEM@mPLV (G5). The mice received intravenous administration of different drugs every 2 days for a total of five treatments (dose of PF = 10 mg/kg and GEM = 5 mg/kg). The treatment outcomes of various groups regarding tumor progression are summarized in Fig. 6 (B to E). Compared to the control group, the other treatment groups significantly inhibited residual tumor growth. On day 22 posttumor inoculation, encapsulation of the drugs within mPLVs led to a further decrease in tumor volume compared to free drugs. Notably, the PF/GEM@mPLV-treated group demonstrated the most pronounced tumor reduction among all groups, with an approximately fourfold decrease in tumor volume relative to the control group (Fig. 6, D and E). The variation trend of tumor weight was consistent with that of tumor volume, as indicated in Fig. 6 (B and C). The PDAC tumors were harvested for further pathological analysis. Hematoxylin and eosin (H&E) staining results showed that the control and PF@mPLV groups exhibited prominent large, deeply stained nuclei. In contrast, the PF/GEM@mPLV group displayed more normal connective tissue structures (fig. S15A). In addition, the PF/GEM@mPLV group showed a significant reduction in marker of proliferation Ki-67–positive areas and an increase in terminal deoxynucleotidyl transferase–mediated deoxyuridine triphosphate nick end labeling (TUNEL)–positive areas, indicating its superior tumor-killing effects (fig. S15, A to C). Histological examination of major organs (heart, liver, spleen, lungs, and kidneys) revealed no obvious abnormalities among the groups during the treatment period (fig. S16). Besides, no significant differences in body weight were observed (fig. S17A). The serum biochemical indicators, including liver and kidney function and cardiac enzymes, were measured. The results showed that the serum levels of alanine aminotransferase (ALT), aspartate aminotransferase (AST), blood urea nitrogen (BUN), creatinine, and creatine kinase (CK) remained within normal ranges (fig. S17, B to F). These results collectively demonstrate the high therapeutic biosafety of the PF/GEM@mPLV treatment.

To elucidate the underlying mechanisms of the antitumor effects triggered by PF/GEM@mPLV, we harvested the tumors for analysis 10 days after the first treatment. Compared to the control groups, TNF-α levels in tumor tissues were not significantly elevated after PF/GEM@mPLV treatment (Fig. 6F). However, IFN-γ concentration was markedly increased (Fig. 6G), indicating a potential activation of antitumor T cell immune responses. Systematic evaluation of changes in intratumoral immune cells was conducted using flow cytometric analysis (fig. S18). Although the total TAM proportion showed no significant decrease compared to the control group (fig. S19, A and B), the proportion of CCR2^+^ TAMs decreased significantly from 34.2% (control) to 15.4% (free PF + GEM), 15.2% (PF@mPLV), and 6.5% (PF/GEM@mPLV), respectively (Fig. 6, H and I). In addition, the immunosuppressive M2-TAMs showed a significant decrease in the PF/GEM@mPLV group, suggesting a repolarization effect induced by the treatment (Fig. 6, J and K). Moreover, the PF/GEM@mPLV treatment resulted in a marked enhancement of CD8^+^ T cells (Fig. 6, L and M) while having minimal impact on CD4^+^ T cells (fig. S19C). mIF demonstrated a significant increase in the proportion of granzyme B^+^CD8^+^ T cells in the PF@mPLV and PF/GEM@mPLV groups compared to the control group, with the highest levels observed in the PF/GEM@mPLV group (Fig. 6, N and O). Although the proportion of T_reg_ cells showed no significant decrease, the CD8^+^ T/T_reg_ cell ratio, an important indicator of antitumor immunity, was markedly elevated after PF/GEM@mPLV treatment (fig. S19, D to F). These findings demonstrate that the synergistic effects of PF/GEM@mPLV treatment effectively modulate the TME by diminishing CCR2^+^ TAM recruitment, repolarizing M2-TAMs, and enhancing the infiltration of cytotoxic T cells [cytotoxic T lymphocytes (CTLs)], thus providing a robust antitumor immune response.

### PF/GEM@mPLV inhibits liver metastasis and elicits antitumor immunity in orthotopic tumors

**Fig. 7.**
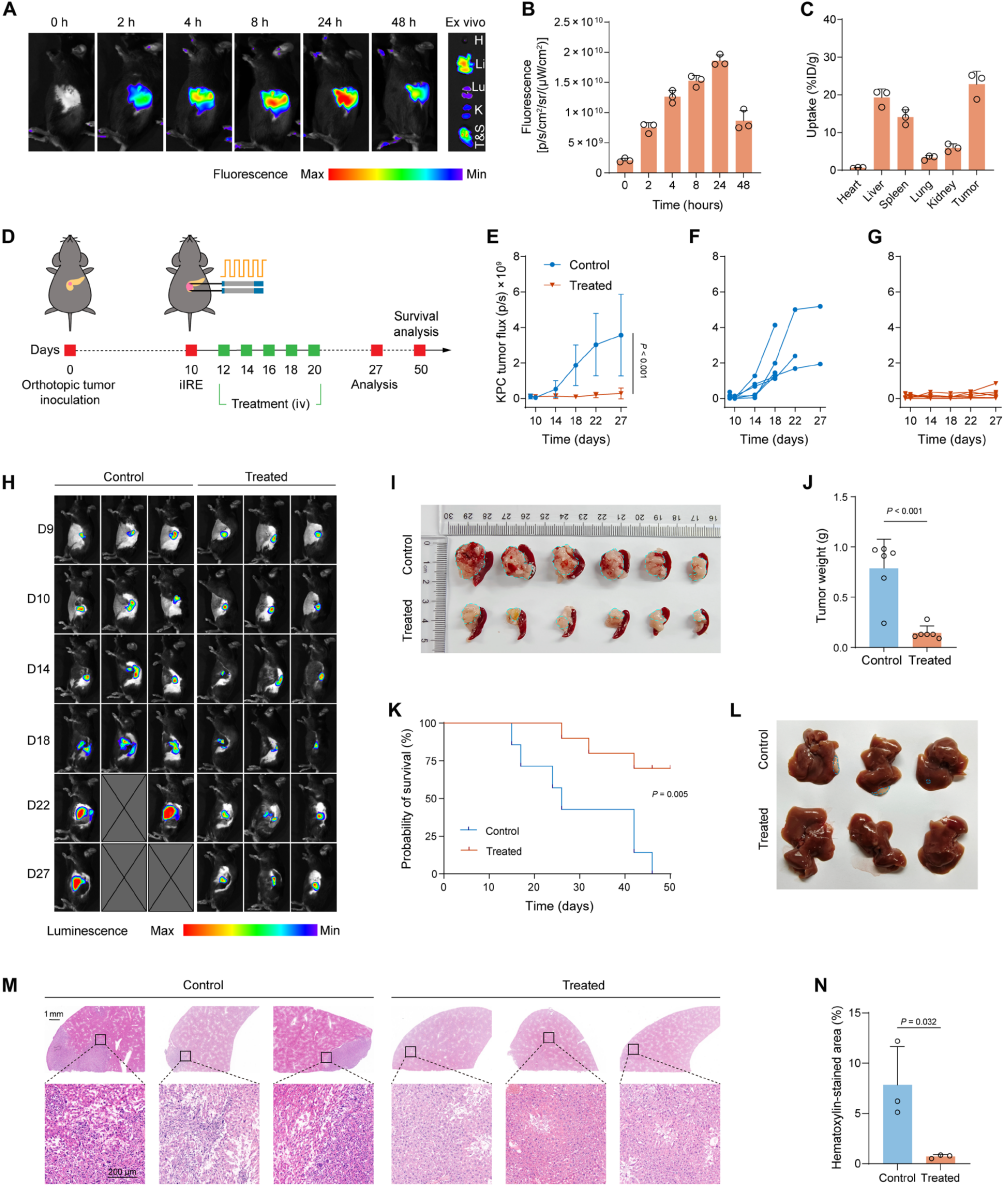
PF/GEM@mPLVs suppress post-iIRE tumor recurrence and liver metastasis in orthotopic KPC tumors. (**A**) Representative in vivo fluorescence images of orthotopic KPC tumor–bearing mice captured at 0, 2, 4, 8, 24, and 48 hours after intravenous administration of Cy5.5-labeled PF/GEM@mPLV. The image on the right shows ex vivo fluorescence images of major organs and tumors (T&S represents tumor and spleen) at 48 hours. (**B**) Quantification of in vivo fluorescence (*n* = 3). (**C**) Quantification of nanoparticle biodistribution in major organs and tumor tissue, expressed as %ID/g, 48 hours after administration (*n* = 3). (**D**) Schematic diagram of the treatment strategy in orthotopic KPC tumors. (**E**) Tumor growth in the orthotopic KPC tumor model measured by bioluminescence imaging (*n* = 6). (**F**) Tumor growth of the control group. (**G**) Tumor growth of the treated group. (**H**) Representative bioluminescence images of orthotopic KPC tumors for monitoring tumor growth in the control and treated groups. D, day. (**I**) Gross images of KPC tumors with spleens. (**J**) Tumor weights in the control and treated groups (*n* = 6). (**K**) Kaplan-Meier survival curves (*n* = 10). (**L**) Gross image of liver metastasis. (**M**) Representative image of H&E staining for liver metastases. Scale bars, 1 mm (1× images) and 200 μm (10× images). (**N**) Quantitative analysis of hematoxylin-stained area (*n* = 3). Data are expressed as mean ± SD. Statistical differences were calculated using Student’s *t* test.

To better mimic the pathogenesis of PDAC, we evaluated the therapeutic effects of PF/GEM@mPLV in mice with orthotopic KPC tumors. Likewise, we assessed the tumor-targeting ability of the Cy5.5-labeled PF/GEM@mPLV nanovesicle in post-iIRE orthotopic tumors. The IVIS results showed a continuous accumulation of the nanovesicle in the orthotopic tumors (Fig. 7, A and B). Ex vivo fluorescence imaging revealed a notable accumulation of PF/GEM@mPLV in the tumor, which was comparable to the enrichment observed in the liver (Fig. 7C). Next, mice with orthotopic tumors were randomly assigned to either the control group or the PF/GEM@mPLV-treated group, using the same dosage and treatment intervals as those in the subcutaneous tumor model (Fig. 7D). Bioluminescence imaging was used to monitor orthotopic tumor growth. Compared to the control group, the treated group exhibited significant inhibition of tumor growth and a significant reduction in tumor weight (Fig. 7, E to J). Notably, PF/GEM@mPLV treatment significantly extended the survival of mice with orthotopic PDAC tumors, with 75% of the mice in the treated group surviving at the 50-day endpoint (Fig. 7K). Analyses of tumor pathology, Ki67, and TUNEL assays further demonstrated the robust antitumor efficacy of PF/GEM@mPLV (fig. S20). In addition, the safety profile of PF/GEM@mPLV was comprehensively assessed in the orthotopic tumor model. Histopathological examination of major organs revealed no abnormalities (fig. S21). Serum biochemical markers remained within the normal range, and no statistically significant alterations were observed in body weight (fig. S22).

The liver is the predominant site for distant metastasis in pancreatic cancer, primarily due to hematogenous dissemination (*44*). Accordingly, we evaluated spontaneous liver metastases in the two groups. Macroscopic examination revealed that nearly all mice in the control group exhibited visible metastatic nodules in the liver, whereas fewer liver metastases were observed in the treated group (Fig. 7L). In the H&E staining of the liver section, metastatic tumors were observed in the control group, whereas micrometastases were nearly absent in the treated group (Fig. 7M). This observation was further supported by semiquantitative analysis (Fig. 7N).

**Fig. 8.**
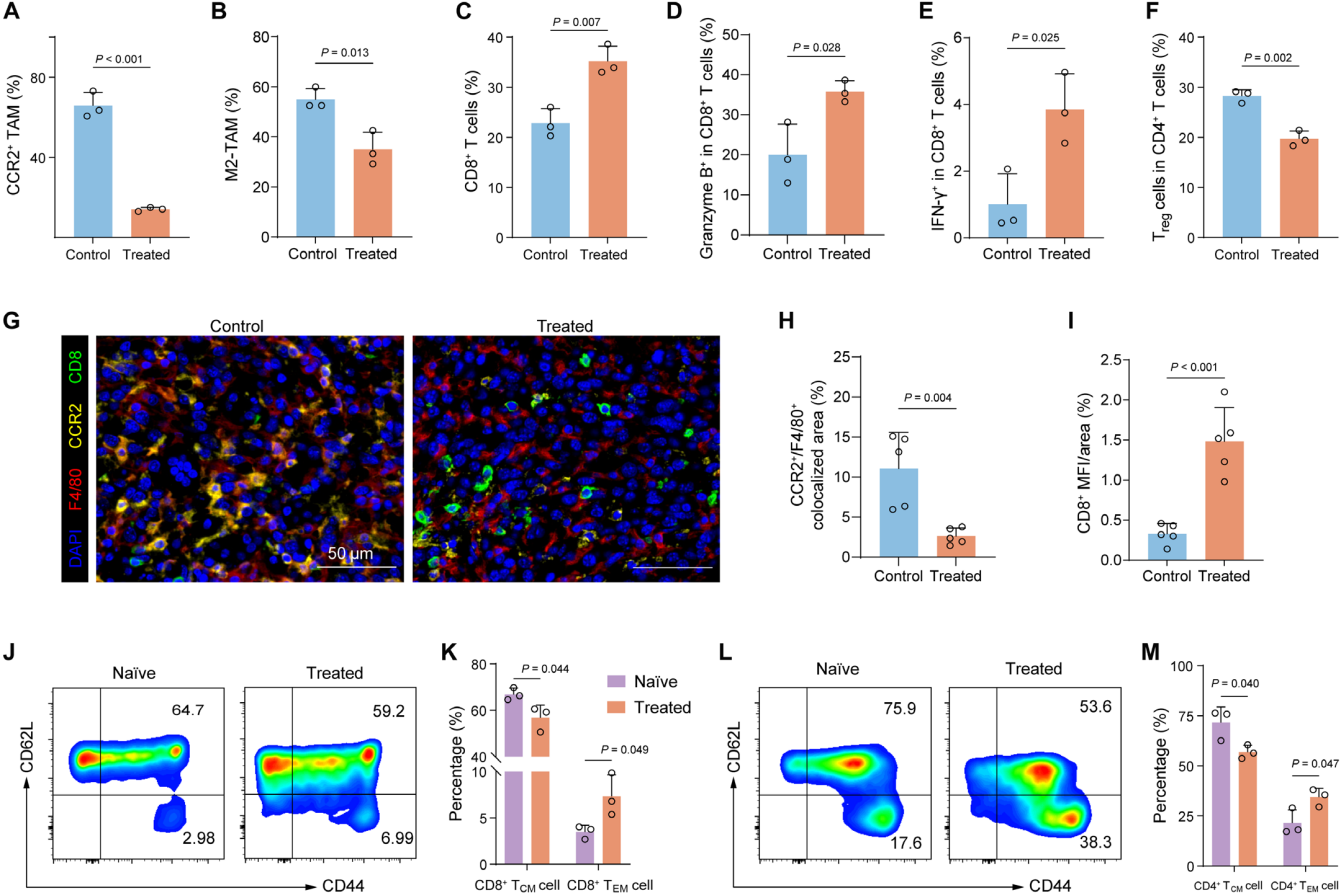
Local and systemic immune responses in orthotopic KPC tumors. (**A** to **F**) Flow cytometric analysis of CCR2^+^ TAM (CCR2^+^Ly6G^−^F4/80^+^CD11b^+^CD45^+^), M2-TAM (CD206^hi^Ly6G^−^F4/80^+^CD11b^+^CD45^+^), CD8^+^ T cell (CD8^+^CD3^+^CD45^+^), granzyme B^+^CD8^+^ T cell (granzyme B^+^CD8^+^CD3^+^CD45^+^), IFN-γ^+^CD8^+^ T cell (IFN-γ^+^CD8^+^CD3^+^CD45^+^), and T_reg_ cell (CD25^+^Foxp3^+^CD4^+^CD3^+^CD45^+^) (*n* = 3). (**G**) Representative mIF images of tumor tissues showing DAPI (blue), F4/80 (red), CCR2 (yellow), and CD8 (green) (scale bars, 50 μm). (**H**) Quantification of CCR2^+^F4/80^+^ colocalized pixels relative to the total area (*n* = 5 FOVs). (**I**) Quantification of CD8^+^ mean fluorescence intensity (MFI) relative to the total area (*n* = 5 FOVs). (**J** to **M**) Representative flow cytometric analysis and quantification of (J and K) CD8^+^ central memory T cell (CD8^+^ T_CM_ cell; CD62L^+^CD44^+^CD8^+^) and CD8^+^ T_EM_ cell (CD62L^−^CD44^+^CD8^+^) subset and (L and M) CD4^+^ T_CM_ cell (CD62L^+^CD44^+^CD4^+^) and CD4^+^T_EM_ cell (CD62L^−^CD44^+^CD4^+^) in naïve and treated mice (*n* = 3). Data are expressed as mean ± SD. Statistical differences were calculated using Student’s *t* test.

We further evaluated the immune microenvironment indicators in the orthotopic PDAC tumor model. Consistent with previous observations, the results demonstrated a decrease in the percentages of CCR2^+^ TAMs, M2-TAMs, and T_reg_ cells within tumors treated with PF/GEM@mPLV, accompanied by an increase in granzyme B^+^ or IFN-γ^+^ CTLs (Fig. 8, A to F). The changes in CCR2^+^ TAMs and CTLs were further confirmed using mIF assays (Fig. 8G). As observed, there was a significant reduction in CCR2^+^F4/80^+^ colocalized areas within the tumor region, accompanied by a notable increase in CD8^+^ areas (Fig. 8, H and I). Moreover, long-term protective immune responses predominantly result from immunological memory (*45*). Upon rechallenge, the effects of immune memory enable the immune system to mount a rapid and robust response to tumor antigens (*46*). To further evaluate whether the PF/GEM@mPLV treatment induces potential immunological memory, we collected the spleens from the treated and healthy control mice (naïve) for subsequent flow cytometry analysis (fig. S23). The results demonstrated that, compared to naïve mice, PF/GEM@mPLV-treated mice exhibited increased levels of effector memory T cells (T_EM_ cells) in both CD4^+^ and CD8^+^ T cells (Fig. 8, J to M). These findings indicate that the long-term immune-memory effects should be elicited by PF/GEM@mPLV treatment.

## DISCUSSION

Clinical and preclinical evidence demonstrates substantial potential for IRE-based therapies in the treatment of pancreatic cancer (*47*, *48*). However, because of the poorly defined tumor margins or undetected micrometastases, PDAC tumors are often incompletely destructed, leading to tumor recurrence (*10*, *11*, *49*). Thus, there is an urgent need to develop a more efficient strategy to enhance the therapeutic benefit of IRE, thereby fully realizing its clinical potential. Here, using a preclinical orthotopic PDAC tumor model, we provide direct evidence that iIRE treatment induces a CCR2^+^ TAM-mediated immunosuppressive microenvironment in the residual tumor, ultimately accelerating tumor progression. Therefore, we rationally designed proteolipid nanovesicles coloaded with the CCR2 antagonist PF-4136309 and GEM for highly efficient tumor combination chemoimmunotherapy. Using both subcutaneous and orthotopic KPC tumor models, our results demonstrated that systemic administration of PF/GEM@mPLV can effectively deliver therapeutic agents to tumors and significantly reverse the immunosuppressive TME in residual PDAC following iIRE therapy. This resulted in a significant inhibition of tumor growth and the development of liver metastases, ultimately leading to prolonged survival in immunocompetent mice with well-established orthotopic PDAC tumors. Thus, this approach represents a promising therapeutic strategy with substantial potential to enhance the clinical outcomes of IRE treatment for pancreatic tumors.

Our results highlight that the combination with IRE and PF/GEM@mPLV could provide an effective clinical solution for locally advanced pancreatic cancer. The unique advantages of the combination treatment are as follows: (i) We first leveraged scRNA-seq, flow cytometry, and multiple immunohistochemical techniques to elucidate the biological effects of IRE on residual tumors. The design of CCR2^+^ TAM–targeted immunotherapeutic combinations was refined according to the tumor immune landscape to overcome these resistance mechanisms. (ii) To enhance the concentration of free therapeutics in tumors, this study used a leukocyte-related drug delivery system. By leveraging the inflammation-targeted and enhanced permeability and retention effect, PF/GEM@mPLV selectively accumulates in both subcutaneous and orthotopic pancreatic tumors following iIRE treatment, thereby enhancing therapeutic efficacy while minimizing systemic toxicity. (iii) We further demonstrated that the PF-induced remodeling of the TME established a foundation for an enhanced response to GEM treatment. All components used in the synthesis of PF/GEM@mPLV are FDA approved except for the macrophage membrane, and their biosafety has been validated, highlighting their potential for clinical translation. This innovative integration of local and systemic therapies could have a profound impact on advanced pancreatic cancer care.

This study has several limitations. We did not explore the impact of the drug administration sequence on treatment efficacy. Whether administering the drug before IRE could enhance therapeutic outcomes remains a question worth exploring. Furthermore, although we used KPC cells to establish tumor models, which closely mimic the pathogenesis of human PDAC, it is unclear whether similar immune cell changes exist in human patients after IRE. Currently, we have only observed the efficacy of the nanovesicle in mouse models. When applying this approach to human patients, obtaining low-immunogenic human macrophage membrane proteins presents a challenge due to ethical concerns and a limited quantity of macrophages.

## MATERIALS AND METHODS

### Materials

Dipalmitoyl phosphatidylcholine (DPPC), 1,2-distearoyl-sn-glycero-3-phosphocholine (DSPC), 1,2-distearoyl-sn-glycero-3-phosphoethanolamine-*N*-[amino(polyethylene glycol)2000] (DSPE-PEG-2000), Cy5.5-DSPE-PEG, and cholesterol were purchased from Xi’an Ruixi Biological Technology Co. Ltd. Membrane protein was extracted by Minute Plasma Membrane Protein Isolation and Cell Fractionation Kit (Invent Biotechnologies Inc.). GEM was purchased from Macklin Inc. PF-4136309 was obtained from Shanghai Yuan Chuang Biotechnology Co. Ltd. Methanol was purchased from Shanghai Aladdin Biochemical Technology Co. Ltd., and trichloromethane (CHCl_3_) was purchased from Jiangsu Kanghong Co. Ltd. CCK-8 was purchased from Abbkine Scientific Co. Ltd. ELISA Kit was purchased from YOBIBIO (Shanghai, China).

### Cell lines and animals

The J774A.1, 4T1, CT26, PANC-1, and KP-4 cell lines were originally obtained from the American Type Culture Collection. FC1199 cell line (derived from KPC mice) was provided by J. Xue (State Key Laboratory of Oncogenes and Related Genes, Stem Cell Research Center, Renji Hospital, School of Medicine, Shanghai Jiao Tong University, Shanghai, P.R. China). All cells were cultured in Dulbecco’s modified Eagle’s medium (YOBIBIO) containing 1% penicillin (YOBIBIO), 1% streptomycin (YOBIBIO), and 10% fetal bovine serum (YOBIBIO) at 37°C in 5% CO_2_ humidified air. The cells were detected every 2 months to exclude mycoplasma. Male C57BL/6 mice (5- to 7-week-old) were ordered from Huachuang Sino Co. Ltd. (Jiangsu, China). Mice were housed in a specific pathogen–free facility with a 12-hour light/dark cycle at ~25°C and a relative humidity of 40 to 70%. All animal experiments were performed according to protocols in accordance with the policies of the National Ministry of Health and approved by the Laboratory Animal Center of Shanghai Tenth People’s Hospital (SHDSYY-2023-Y3471). The maximum tumor volume allowed by the Experimental Animal Center of Shanghai Tenth People’s Hospital is 1500 mm^3^, and the maximum tumor diameter allowed by the National Institutes of Health is not to exceed 2 cm (Guidelines for Endpoints in Animal Study Proposals). The tumor volume or diameter of the mice in the tumor-related animal experiments in this study did not exceed the allowed range described above.

### Construction of animal models

To establish subcutaneous models, KPC cells (1 × 10^6^) resuspended in 100 μl of PBS were subcutaneously injected into the right flank of male C57BL/6 mice (5- to 7-week-old). To establish orthotopic models, C57BL/6 mice were anesthetized with pentobarbital sodium (1%; 100 mg/kg) and isoflurane [2% at fresh gas flow (4 liters/min)], and a 0.5-cm incision was made in the left abdomen to expose the pancreas. KPC cells (1 × 10^6^) resuspended in 40 μl of cold PBS/Matrigel (Corning) (1:1, v:v) were injected into the pancreatic tail. Mice were monitored with ultrasound imaging (GE HealthCare, Milwaukee, WI, USA) to validate the implantation of orthotopic tumors (fig. S2).

To establish iIRE tumor models, IRE was operated when tumor size reached 7 to 9 mm (about 9 to 10 days after tumor inoculation) using a square electroporator (Hangzhou Ruidi Biotechnology Ltd., SPA-C02). The two needles were inserted perpendicularly to the long axis of the tumor, ablating part of the tumor. The treatment parameters were as follows: two needles with a 5-mm interval, voltage: 1000 V, pulse width: 100 μs, frequency: 1 Hz, and number of repeated pulses: 99. Sham surgery was performed by inserting the same needle in the control mouse model.

Mice were randomly assigned to the experimental groups, and their body weights were monitored every 2 days. Tumor size was calculated using the formula: 1/2 × length × width^2^. For experiments requiring monitoring of the orthotopic tumor, a luciferase-labeled KPC cell line was established and injected into the pancreas with cell numbers of 1 × 10^6^ per mouse. Bioluminescence was detected using the AniView100 Multi-mode In Vivo Animal Imaging System (Guangzhou Biolight Biotechnology Co. Ltd.).

### Imaging examination

Conventional ultrasound: Mice were anesthetized and scanned using a GE LOGIQ E9 scanner (GE HealthCare, Milwaukee, WI, USA) with a 15-MHz linear transducer. CEUS: Contrast agent (SonoVue, Bracco, Milan, Italy) was administered via tail vein injection at a dose of 100 μl per mouse. Real-time CEUS imaging was acquired with a 9-MHz linear transducer under a low mechanical index (MI = 0.13) mode to observe the enhancement pattern within and around the tumor.

### Single-cell RNA sequencing

Tumor tissues, representing the control group and the iIRE group, were excised and immediately processed to obtain a single-cell suspension. Single-cell sequencing libraries were constructed using the Chromium Single Cell 5′ Library Construction Kit v2 (10x Genomics). The libraries were sequenced on an Illumina NovaSeq 6000 platform to generate paired-end reads. The sequencing and analysis were performed by OE Biotech Co. Ltd. (Shanghai, China).

### scRNA-seq data processing

Sequencing data were processed by the Cell Ranger software pipeline (version 3.1.0). Raw sequencing reads were demultiplexed and aligned to the mouse reference genome (mm10) to generate feature-barcode matrices. Quality control was performed to exclude cells with low UMI counts, high mitochondrial gene content, or doublets following the criteria: gene numbers < 200, UMI < 1000, log_10_GenesPerUMI < 0.7, proportion of UMIs mapped to mitochondrial genes > 5%, and proportion of UMIs mapped to hemoglobin genes > 5%. In addition, doublets were removed using the DoubletFinder software (v2.0.2). Further quality control and processing were performed using the Seurat package (version 3.0). Principal components analysis (PCA) was used for linear dimensionality reduction based on gene expression levels. The PCA results were visualized in two-dimensional space using UMAP (Uniform Manifold Approximation and Projection) for nonlinear dimensionality reduction. Marker gene identification was performed using the FindAllMarkers function from the Seurat package, which identifies genes that are differentially up-regulated in each cell type compared to other cell groups. Clustering was performed using the Shared Nearest Neighbor (SNN) algorithm, resulting in optimal cell groupings. The identified marker genes were visualized using the VlnPlot and FeaturePlot functions. Differential gene expression analysis was performed using the FindMarkers function from the Seurat package. Genes with *P* < 0.05 and fold change >1.2 were considered significant DEGs. Significant DEGs were then subjected to GO and Kyoto Encyclopedia of Genes and Genomes pathway enrichment analysis.

### Reclustering of macrophage

Macrophage cells were reclustered to further investigate macrophage subtypes. High variability genes (HVGs) were reselected for the macrophage subtypes as previously described. PCA was applied to the selected HVGs for dimensionality reduction. The cells were then reclustered using the SNN algorithm and visualized in two-dimensional space using UMAP as described above.

### mIF staining

mIF was performed on 5-μm tissue sections from mouse PDAC tumors. F4/80/CCR2/CD8 assay used the four-color multiplex fluorescence immunohistochemical staining kit (abs50028, Absin) according to the manufacturer’s instructions. Slides were blocked with 5% blocking buffer (goat serum diluted in tris-buffered saline with Tween 20) before incubating with primary antibodies at room temperature (RT). The primary antibodies used and their specific conditions were as follows: CCR2: Anti-mouse CCR2 (1:200; Abcam, ab273050) was incubated overnight at 4°C. Antigen retrieval was performed using tris buffer. The slides were stained with Opal 570 (yellow) for 10 min at RT. F4/80: Anti-mouse F4/80 (1:300; Cell Signaling Technologies, 70076) was incubated at RT for 1 hour. Antigen retrieval was performed using washing buffer. The slides were stained with Opal 650 (red) for 10 min at RT. CD8: Anti-mouse CD8 (1:200; Abcam, ab217344) was incubated overnight at 4°C. Antigen retrieval was performed using washing buffer. The slides were stained with Opal 520 (green) for 10 min at RT. Nuclei were stained with 4′,6-diamidino-2-phenylindole (DAPI) (1:100; Absin, abs47047616) for 10 min, followed by washing and mounting with mounting medium. Granzyme B/CD8 assay followed a similar procedure, using the following primary antibodies: Anti-mouse CD8 (1:200; Abcam, ab217344am) and anti-mouse granzyme B (1:300; Abcam, ab255598). Slides were scanned using Pannoramic MIDI II digital slide scanners (3DHISTECH Ltd.). Positive areas were quantified using ImageJ software.

### Quantitative PCR

Tumor tissues were assessed 3 days after iIRE treatment. Total RNA was extracted using FreeZol Reagent (Vazyme, R711-01), and cDNA was synthesized using HiScript III RT SuperMix for qPCR (+gDNA wiper) (Vazyme, R323-01). qPCR was performed using ChamQ Blue Universal SYBR qPCR Master Mix (Vazyme, Q312-02) on a LightCycler 96 System (Roche, 05815916001). The relative expression was calculated using the 2^–ΔΔCT^ method. Primers: mouse *Ccr2* (forward, 5′-TTTGTTTTTGCAGATGATTCAA-3′; reverse, 5′-TGCCATCATAAAGGAGCCAT-3′) and mouse *Gapdh* (forward, 5′-AAGAAGGTGGTGAAGCAGGC-3′; reverse, 5′-TCCACCACCCTGTTGCTGTA-3′).

### Flow cytometry and FACS

PDAC tumors or spleens were digested into single-cell suspensions and resuspended in cell staining buffer. CD16/CD32 monoclonal antibody (eBioscience, FRC-4G8, MFCR00) was used to block nonspecific binding of FcγRIII/II. Fixable Viability Stain 450 (BD Biosciences, 562247) or Fixable Viability Dye eFluor 780 (Thermo Fisher Scientific, 65-0865-14) was used to stain dead cells. For cell surface antigens, cells were stained with mouse antibodies. For intracellular antigens, cells were fixed and permeabilized, followed by staining with mouse antibodies. Antibodies used in this study included the following: CD45-Brilliant Violet 510 (BioLegend, 103138), CD11b–fluorescein isothiocyanate (FITC; Thermo Fisher Scientific, 11-0112-82), F4/80-allophycocyanin (APC; Thermo Fisher Scientific, 17-4801-82), Ly6G–eFluor 450 (Thermo Fisher Scientific, 48-9668-82), CD45–Alexa Fluor 700 (BioLegend, 103128), F4/80-BV650 (BioLegend, 123149), LYVE1 (Abcam, ab281587), C1q-Brilliant Blue 700 (BB700; BD Biosciences, 756678), CCR2–phycoerythrin (PE)–CD192 (BioLegend, 150610), CD3-BV421 (BD Biosciences, 562600), CD8-PerCP-Cy5.5 (BioLegend, 100734), CD69-FITC (BioLegend, 104506), IFN-γ–PE (BioLegend, 505808), granzyme B–APC (BioLegend, 372204), granzyme B–PE (BioLegend, 396406), CD4-FITC (Thermo Fisher Scientific, 11-0042-85), CD25-APC (Thermo Fisher Scientific, 17-0251-82), CD44-PE (Thermo Fisher Scientific, 12-0441-82), CD62L-APC (Thermo Fisher Scientific, 17-0621-82), CD206-PE-Cy7 (BioLegend, 141720), and forkhead box P3 (Foxp3)–PE (Thermo Fisher Scientific, 12-5773-82). Secondary antibodies included APC (Abcam, ab130805). Flow cytometry was performed on the LSRFortessa X-20 Cell Analyzer (BD Biosciences). FACS was performed on FACSAria III (BD Biosciences). The data were analyzed using FlowJo software (TreeStar, 10.6.2).

### T cell assay

CD8^+^ T cells were isolated from mouse spleen with anti-CD8 magnetic beads (STEMCELL, 17853) and then stimulated with CD3/CD28 T Cell Activator (STEMCELL, 10971) and recombinant interleukin-2 (IL-2; R&D Systems, USA). LYVE1^+^, C1q^+^, or CCR2^+^ TAMs were sorted from KPC tumor 3 days after iIRE. For T cell proliferation assay, CD8^+^ T cells were labeled with carboxyfluorescein diacetate succinimidyl ester (CFSE; Invitrogen) and cocultured with TAMs for 5 days (CD8^+^ T cells: TAM = 1 × 10^5^:1 × 10^4^). For T cell cytotoxicity assay, CD8^+^ T cells were cocultured with ConA and TAMs for 5 days as well. Control groups included CD8^+^ T cells cultured without ConA or TAMs. CFSE signals and cell markers CD69, IFN-γ, and granzyme B on T cells were assessed by flow cytometry.

### Synthesis of PF/GEM@mPLV

PF/GEM@mPLV was synthesized on the basis of the thin-film evaporation method. Briefly, DPPC, DSPC, DSPE-PEG-2000, and cholesterol (mass ratio of 3:1:1:1) with a total lipid amount of 50 mg, PF and GEM 5 mg each, were dissolved in a chloroform and methanol mixture [3:1 (v/v)]. Then, the solvent was evaporated using a rotary evaporator at 60°C, 100 rpm, and 100 mbar to form a lipid film. The lipid film was hydrated with either PBS alone or PBS containing membrane proteins (protein to lipid mass ratio of 1:300) to assemble liposomes or mPLV, respectively. Membrane proteins were extracted from the immortalized mouse cell line J774A.1 using the INVENT Membrane Protein Extraction Kit SM-005, and protein quantification was performed using the bicinchoninic acid (BCA) assay. The lipid suspensions were extruded through cellulose acetate membranes with pore sizes of 450 and 200 nm, followed by dialysis overnight through a 1000-kDa dialysis membrane to remove free drugs and proteins.

### Characterizations

TEM (FEI, Tecnai G2 F30) was used to observe the morphology and structures of nanovesicles. DLS (Zetasizer Nano ZS90) was used to measure the particle size and zeta potential of the nanovesicles. The stability of the nanovesicles was assessed by measuring the mean particle size in PBS (pH 7.4) at different time points. The membrane proteins loaded onto the nanovesicles were detected using an SDS-PAGE assay. To measure the encapsulation efficiency (EE%) and drug loading capacity (DLC%), the liposome suspension was dissolved in methanol and subjected to ultrasonic disruption. The concentration of GEM was measured using UV-vis spectrophotometry (Shimadzu, UV-3101PC spectroscope) at 269 nm. The concentration of PF was measured at 245 nm using HPLC (Waters, E2695) at the following testing conditions: Shimadzu Shimpack GIST C18 column (4.6 mm by 250 mm, 5 μm), 0.1% phosphoric acid in water (Mobile Phase A) and 100% acetonitrile (Mobile Phase B), and gradient elution program. Calculation formulas

EE% = (amount of drug encapsulated/total amount of drug added) × 100%

DLC% = [amount of drug encapsulated/(total amount of lipid + drug)] × 100%

For the in vitro drug release studies, freshly synthesized PF/GEM@mPLV was dissolved in 1 ml of PBS solution and placed into a dialysis bag (molecular weight cutoff: 3500 kDa). The dialysis bag was then immersed in 30 ml of PBS solution containing 0.2% Tween 20 and incubated in a 37°C shaker. At the indicated time points (0.5, 3, 12, 24, 48, and 72 hours), the drug concentration in the dialysis bag was measured.

### Cellular uptake assay

1 × 10^5^ to 2 × 10^5^ macrophages (J774A.1) or KPC cells were seeded into six-well plates and cultured at 37°C with 5% CO_2_ for 24 hours. Subsequently, the cells were incubated with DiI-labeled liposome/mPLV at 37°C with 5% CO_2_ for 2, 4, and 8 hours, respectively. The cells were then fixed with 4% paraformaldehyde. The nuclei were stained with DAPI, and the cells were observed under a confocal laser scanning microscope (CLSM).

### In vitro cytotoxicity assay

The effect of the drug on KPC cells was assessed using the CCK-8 assay. Cells were seeded into 96-well plates (1 × 10^4^ cells per well) and cultured overnight. The following day, the cells were treated with different concentrations of GEM, diluted in cell culture medium to a series of concentrations (3.12, 6.25, 12.5, 25, 50, 100, and 200 ng/ml), with three replicates for each concentration. After 24 hours of drug treatment, cell viability was measured by the standard protocol of the CCK-8 assay.

### Cytokine detection and CM preparation

The concentration of cytokine CCL2 in different cell lines (J774A.1, KPC, 4T1, CT26, PANC-1, and KP-4) was detected using standard mouse or human ELISA Kit protocol. The KPC CM was collected from cell supernatants after 48 hours of culture, centrifuged to remove cell debris, sterilized using a 0.22-μm filter, and stored at −20°C.

### Calcium influx assay

5 × 10^4^ J774A.1 macrophages were seeded on 3.5-cm confocal dishes and incubated overnight. After loading with Fluo-4 AM at 37°C for 30 min, the cells were treated with different formulations for 1 hour, with or without CM for an additional 30 min. Intracellular calcium levels were then visualized at ×40 magnification using CLSM and quantified by ImageJ software.

### Macrophage transwell migration assay

Two hundred microliters of serum-free medium containing 1 × 10^5^ macrophages was seeded into the upper chamber of a transwell insert pores (8 μm; Corning, 3422) in a 24-well plate. The lower chamber was filled with 600 μl of CM (previously incubated for 48 hours) as the chemoattractant. After a 12-hour incubation, cells in the upper chamber were carefully removed. The cells that migrated through the membrane were fixed with 4% paraformaldehyde and stained with 1% crystal violet. Following washing with distilled water, stained cells were counted in five randomly selected fields of view under a microscope at ×20 magnification. The number of migrated cells was quantified by ImageJ software.

### In vivo and ex vivo fluorescence imaging

Mice after treatment with iIRE were administered with 100 μl of Cy5.5-labeled PF/GEM@Liposome or PF/GEM@mPLV via the tail vein. The distribution of the fluorescently labeled materials was monitored after anesthetizing the mice with isoflurane at 0, 2, 4, 8, 24, and 48 hours postinjection using the IVIS system (Guangzhou Biolight Biotechnology Co., Ltd.). At the 48-hour time point, the mice were euthanized, and major organs including the heart, liver, spleen, lungs, and kidneys were harvested to assess the biodistribution of the materials. The average fluorescence intensity and tissue uptake were quantified for each group. For microscopic analysis of the intratumoral distribution, PF/GEM@Liposome or PF/GEM@mPLV was injected into post-iIRE mice. The tumors were harvested at 24 hours, rapidly frozen in liquid nitrogen, embedded in optimal cutting temperature compound, and sectioned for fluorescence imaging.

### Antitumor effect in subcutaneous and orthotopic tumors

The construction of subcutaneous and orthotopic recurrent tumor models was performed as described above. For the subcutaneous model, mice were randomly divided into five groups for different treatments and were intravenously injected with PBS solution (100 μl), free PF + GEM (100 μl; PF = 10 mg/kg and GEM = 5 mg/kg), PF@mPLV (100 μl; PF = 10 mg/kg), GEM@mPLV (100 μl; GEM = 5 mg/kg), or PF/GEM@mPLV (100 μl; PF = 10 mg/kg and GEM = 5 mg/kg), respectively. Treatments were performed on days 12, 14, 16, 18, and 20 for a total of five sessions. When one of the tumor volumes reached 1500 mm^3^ (treatment endpoint), mice were euthanized, and blood, tumors, and major organs were harvested for further analysis. Tumor weight was recorded at endpoint. To evaluate the biosafety of the treatments, serum biochemical parameters were measured including liver function indicators (ALT and AST), kidney function indicators (BUN and creatinine), and CK. Histopathological analysis of major organs (heart, liver, spleen, lungs, and kidneys) was performed to detect tissue-level toxicity or abnormalities using H&E staining. ELISA, flow cytometry, and mIF were performed to evaluate intratumoral cytokines and immune cells.

For the orthotopic model, mice were randomly divided into a control (PBS) and a treated group (PF/GEM@mPLV). Treatments were performed on days 12, 14, 16, 18, and 20. At the indicated time points, the growth of orthotopic PDAC tumors was monitored using bioluminescence imaging with the IVIS system. H&E staining and a serum biochemical test were performed in the orthotopic model, too. The survival endpoint for tumor-bearing mice was determined on the basis of the onset of moribund status, inability to move, or when the tumor volume reached 1500 mm^3^. The survival rate of the mice was recorded and plotted as Kaplan-Meier survival curves. Liver metastases were examined by H&E staining. Flow cytometry and mIF were performed to evaluate intratumoral immune cells.

### Statistical analysis

All quantitative data are expressed as mean ± SD. Statistical significance between groups was determined using Student’s *t* test for two-group comparison or one-way analysis of variance (ANOVA) and two-way ANOVA for multiple-group comparison by GraphPad Prism software (version 9.0.0), as stated in the figure legends. Survival data were analyzed by log-rank test using GraphPad Prism. *P* < 0.05 was considered significant.

## References

[1] F. Bray, M. Laversanne, H. Y. A. Sung, J. Ferlay, R. L. Siegel, I. Soerjomataram, A. Jemal, Global cancer statistics 2022: GLOBOCAN estimates of incidence and mortality worldwide for 36 cancers in 185 countries. CA Cancer J. Clin. 74, 229–263 (2024). 38572751 10.3322/caac.21834

[2] M. A. Tempero, M. P. Malafa, M. Al-Hawary, S. W. Behrman, A. B. Benson, D. B. Cardin, E. G. Chiorean, V. Chung, B. Czito, M. Del Chiaro, M. Dillhoff, T. R. Donahue, E. Dotan, C. R. Ferrone, C. Fountzilas, J. Hardacre, W. G. Hawkins, K. Klute, A. H. Ko, J. W. Kunstman, N. LoConte, A. M. Lowy, C. Moravek, E. K. Nakakura, A. K. Narang, J. Obando, P. M. Polanco, S. Reddy, M. Reyngold, C. Scaife, J. Shen, C. Vollmer, R. A. Wolff, B. M. Wolpin, B. Lynn, G. V. George, Pancreatic adenocarcinoma, version 2.2021, NCCN Clinical Practice Guidelines in Oncology. J. Natl. Compr. Canc. Netw. 19, 439–457 (2021). 33845462 10.6004/jnccn.2021.0017

[3] E. M. Stoffel, R. E. Brand, M. Goggins, Pancreatic cancer: Changing epidemiology and new approaches to risk assessment, early detection, and prevention. Gastroenterology 164, 752–765 (2023). 36804602 10.1053/j.gastro.2023.02.012 PMC10243302

[4] A. J. Grossberg, L. C. Chu, C. R. Deig, E. K. Fishman, W. L. Hwang, A. Maitra, D. L. Marks, A. Mehta, N. Nabavizadeh, D. M. Simeone, C. D. Weekes, C. R. Thomas Jr., Multidisciplinary standards of care and recent progress in pancreatic ductal adenocarcinoma. CA Cancer J. Clin. 70, 375–403 (2020). 32683683 10.3322/caac.21626 PMC7722002

[5] M. Suker, B. R. Beumer, E. Sadot, L. Marthey, J. E. Faris, E. A. Mellon, B. F. El-Rayes, A. Wang-Gillam, J. Lacy, P. J. Hosein, S. Y. Moorcraft, T. Conroy, F. Hohla, P. Allen, J. Taieb, T. S. Hong, R. Shridhar, I. Chau, C. H. van Eijck, B. G. Koerkamp, FOLFIRINOX for locally advanced pancreatic cancer: A systematic review and patient-level meta-analysis. Lancet Oncol. 17, 801–810 (2016). 27160474 10.1016/S1470-2045(16)00172-8 PMC5527756

[6] E. P. Balaban, P. B. Mangu, A. A. Khorana, M. A. Shah, S. Mukherjee, C. H. Crane, M. M. Javle, J. R. Eads, P. Allen, A. H. Ko, A. Engebretson, J. M. Herman, J. H. Strickler, A. B. Benson, S. Urba, N. S. Yee, Locally advanced, unresectable pancreatic cancer: American Society of Clinical Oncology Clinical Practice Guideline. J. Clin. Oncol. 34, 2654–2668 (2016). 27247216 10.1200/JCO.2016.67.5561

[7] D. P. S. Sohal, E. B. Kennedy, P. Cinar, T. Conroy, M. S. Copur, C. H. Crane, I. Garrido-Laguna, M. W. Lau, T. Johnson, S. Krishnamurthi, C. Moravek, E. M. O’Reilly, P. A. Philip, S. Pant, M. A. Shah, V. Sahai, H. E. Uronis, N. Zaidi, D. Laheru, Metastatic pancreatic cancer: ASCO guideline update. J. Clin. Oncol. 38, 3217–3230 (2020). 32755482 10.1200/JCO.20.01364 PMC12974607

[8] R. V. Davalos, I. L. Mir, B. Rubinsky, Tissue ablation with irreversible electroporation. Ann. Biomed. Eng. 33, 223–231 (2005). 15771276 10.1007/s10439-005-8981-8

[9] B. E. Bulvik, N. Rozenblum, S. Gourevich, M. Ahmed, A. V. Andriyanov, E. Galun, S. N. Goldberg, Irreversible electroporation versus radiofrequency ablation: A comparison of local and systemic effects in a small-animal model. Radiology 280, 413–424 (2016). 27429143 10.1148/radiol.2015151166

[10] R. C. Martin II, D. Kwon, S. Chalikonda, M. Sellers, E. Kotz, C. Scoggins, K. M. McMasters, K. Watkins, Treatment of 200 locally advanced (stage III) pancreatic adenocarcinoma patients with irreversible electroporation: Safety and efficacy. Ann. Surg. 262, 486–494 (2015). 26258317 10.1097/SLA.0000000000001441

[11] M. D. Kluger, I. Epelboym, B. A. Schrope, K. Mahendraraj, E. M. Hecht, J. Susman, J. L. Weintraub, J. A. Chabot, Single-institution experience with irreversible electroporation for T4 pancreatic cancer: First 50 patients. Ann. Surg. Oncol. 23, 1736–1743 (2016). 26714959 10.1245/s10434-015-5034-x

[12] A. H. Ruarus, L. G. P. H. Vroomen, B. Geboers, E. van Veldhuisen, R. S. Puijk, S. Nieuwenhuizen, M. G. Besselink, B. M. Zonderhuis, G. Kazemier, T. D. de Gruijl, K. P. van Lienden, J. J. J. de Vries, H. J. Scheffer, M. R. Meijerink, Percutaneous irreversible electroporation in locally advanced and recurrent pancreatic cancer (PANFIRE-2): A multicenter, prospective, single-arm, phase II study. Radiology 294, 212–220 (2020). 31687922 10.1148/radiol.2019191109

[13] M. M. Holland, N. Bhutiani, E. J. Kruse, M. J. Weiss, J. D. Christein, R. R. White, K. W. Huang, R. C. G. Martin II, A prospective, multi-institution assessment of irreversible electroporation for treatment of locally advanced pancreatic adenocarcinoma: Initial outcomes from the AHPBA pancreatic registry. HPB 21, 1024–1031 (2019). 30737097 10.1016/j.hpb.2018.12.004

[14] R. C. Martin II, K. McFarland, S. Ellis, V. Velanovich, Irreversible electroporation in locally advanced pancreatic cancer: Potential improved overall survival. Ann. Surg. Oncol. 20, 443–449 (2013). 10.1245/s10434-012-2736-1 23128941

[15] J. Zhao, X. Wen, L. Tian, T. Li, C. Xu, X. Wen, M. P. Melancon, S. Gupta, B. Shen, W. Peng, C. Li, Irreversible electroporation reverses resistance to immune checkpoint blockade in pancreatic cancer. Nat. Commun. 10, 899 (2019). 30796212 10.1038/s41467-019-08782-1 PMC6385305

[16] H. Peng, J. Shen, X. Long, X. Zhou, J. Zhang, X. Xu, T. Huang, H. Xu, S. Sun, C. Li, P. Lei, H. Wu, J. Zhao, Local release of TGF-β inhibitor modulates tumor-associated neutrophils and enhances pancreatic cancer response to combined irreversible electroporation and immunotherapy. Adv. Sci. 9, 2105240 (2022). 10.1002/advs.202105240 PMC8981446 35128843

[17] X. Long, A. Dai, T. Huang, W. Niu, L. Liu, H. Xu, T. Yin, T. Jiang, S. Sun, P. Lei, C. Li, X. Zhu, J. Zhao, Simultaneous delivery of dual inhibitors of DNA damage repair sensitizes pancreatic cancer response to irreversible electroporation. ACS Nano 17, 12915–12932 (2023). 37352467 10.1021/acsnano.3c05009

[18] S. Li, C. Zhu, X. Zhou, L. Chen, X. Bo, Y. Shen, X. Guan, X. Han, D. Shan, L. Sun, Y. Chen, H. Xu, W. Yue, Engineering ROS-responsive bioscaffolds for disrupting myeloid cell-driven immunosuppressive niche to enhance PD-L1 blockade-based postablative immunotherapy. Adv. Sci. 9, e2104619 (2022). 10.1002/advs.202104619 PMC9008797 35156339

[19] Y. Shen, L. Chen, X. Guan, X. Han, X. Bo, S. Li, L. Sun, Y. Chen, W. Yue, H. Xu, Tailoring chemoimmunostimulant bioscaffolds for inhibiting tumor growth and metastasis after incomplete microwave ablation. ACS Nano 15, 20414–20429 (2021). 34881574 10.1021/acsnano.1c08826

[20] X. Guan, L. Sun, Y. Shen, F. Jin, X. Bo, C. Zhu, X. Han, X. Li, Y. Chen, H. Xu, W. Yue, Nanoparticle-enhanced radiotherapy synergizes with PD-L1 blockade to limit post-surgical cancer recurrence and metastasis. Nat. Commun. 13, 2834 (2022). 35595770 10.1038/s41467-022-30543-w PMC9123179

[21] N. Caronni, F. La Terza, F. M. Vittoria, G. Barbiera, L. Mezzanzanica, V. Cuzzola, S. Barresi, M. Pellegatta, P. Canevazzi, G. Dunsmore, C. Leonardi, E. Montaldo, E. Lusito, E. Dugnani, A. Citro, M. S. F. Ng, M. Schiavo Lena, D. Drago, A. Andolfo, S. Brugiapaglia, A. Scagliotti, A. Mortellaro, V. Corbo, Z. Liu, A. Mondino, P. Dellabona, L. Piemonti, C. Taveggia, C. Doglioni, P. Cappello, F. Novelli, M. Iannacone, L. G. Ng, F. Ginhoux, S. Crippa, M. Falconi, C. Bonini, L. Naldini, M. Genua, R. Ostuni, IL-1β^+^ macrophages fuel pathogenic inflammation in pancreatic cancer. Nature 623, 415–422 (2023). 37914939 10.1038/s41586-023-06685-2

[22] Y. Zhu, J. M. Herndon, D. K. Sojka, K. W. Kim, B. L. Knolhoff, C. Zuo, D. R. Cullinan, J. Luo, A. R. Bearden, K. J. Lavine, W. M. Yokoyama, W. G. Hawkins, R. C. Fields, G. J. Randolph, D. G. DeNardo, Tissue-resident macrophages in pancreatic ductal adenocarcinoma originate from embryonic hematopoiesis and promote tumor progression. Immunity 47, 597 (2017). 28930665 10.1016/j.immuni.2017.08.018 PMC5664180

[23] E. F. Davis-Marcisak, A. Deshpande, G. L. Stein-O’Brien, W. J. Ho, D. Laheru, E. M. Jaffee, E. J. Fertig, L. T. Kagohara, From bench to bedside: Single-cell analysis for cancer immunotherapy. Cancer Cell 39, 1062–1080 (2021). 34329587 10.1016/j.ccell.2021.07.004 PMC8406623

[24] Y. Binenbaum, E. Fridman, Z. Yaari, N. Milman, A. Schroeder, G. Ben David, T. Shlomi, Z. Gil, Transfer of miRNA in macrophage-derived exosomes induces drug resistance in pancreatic adenocarcinoma. Cancer Res. 78, 5287–5299 (2018). 30042153 10.1158/0008-5472.CAN-18-0124

[25] J. B. Mitchem, D. J. Brennan, B. L. Knolhoff, B. A. Belt, Y. Zhu, D. E. Sanford, L. Belaygorod, D. Carpenter, L. Collins, D. Piwnica-Worms, S. Hewitt, G. M. Udupi, W. M. Gallagher, C. Wegner, B. L. West, A. Wang-Gillam, P. Goedegebuure, D. C. Linehan, D. G. DeNardo, Targeting tumor-infiltrating macrophages decreases tumor-initiating cells, relieves immunosuppression, and improves chemotherapeutic responses. Cancer Res. 73, 1128–1141 (2013). 23221383 10.1158/0008-5472.CAN-12-2731 PMC3563931

[26] C. J. Halbrook, C. Pontious, I. Kovalenko, L. Lapienyte, S. Dreyer, H. J. Lee, G. Thurston, Y. Zhang, J. Lazarus, P. Sajjakulnukit, H. S. Hong, D. M. Kremer, B. S. Nelson, S. Kemp, L. Zhang, D. Chang, A. Biankin, J. Shi, T. L. Frankel, H. C. Crawford, J. P. Morton, M. Pasca di Magliano, C. A. Lyssiotis, Macrophage-released pyrimidines inhibit gemcitabine therapy in pancreatic cancer. Cell Metab. 29, 1390–1399.e6 (2019). 30827862 10.1016/j.cmet.2019.02.001 PMC6602533

[27] B. Ren, M. Cui, G. Yang, H. Wang, M. Feng, L. You, Y. Zhao, Tumor microenvironment participates in metastasis of pancreatic cancer. Mol. Cancer 17, 108 (2018). 30060755 10.1186/s12943-018-0858-1 PMC6065152

[28] J. P. Neoptolemos, J. Kleeff, P. Michl, E. Costello, W. Greenhalf, D. H. Palmer, Therapeutic developments in pancreatic cancer: Current and future perspectives. Nat. Rev. Gastro. Hepat. 15, 332–347 (2018). 10.1038/s41575-018-0005-x 29717230

[29] T. R. Cox, The matrix in cancer. Nat. Rev. Cancer 21, 217–238 (2021). 33589810 10.1038/s41568-020-00329-7

[30] R. M. Brock, N. Beitel-White, R. V. Davalos, I. C. Allen, Starting a fire without flame: The induction of cell death and inflammation in electroporation-based tumor ablation strategies. Front. Oncol. 10, 1235 (2020). 32850371 10.3389/fonc.2020.01235 PMC7399335

[31] C. He, X. Huang, Y. Zhang, X. Lin, S. Li, T-cell activation and immune memory enhancement induced by irreversible electroporation in pancreatic cancer. Clin. Transl. Med. 10, e39 (2020). 32508058 10.1002/ctm2.39 PMC7403705

[32] R. Molinaro, C. Corbo, J. O. Martinez, F. Taraballi, M. Evangelopoulos, S. Minardi, I. K. Yazdi, P. Zhao, E. De Rosa, M. B. Sherman, A. De Vita, N. E. Toledano Furman, X. Wang, A. Parodi, E. Tasciotti, Biomimetic proteolipid vesicles for targeting inflamed tissues. Nat. Mater. 15, 1037–1046 (2016). 27213956 10.1038/nmat4644 PMC5127392

[33] T. Huang, X. Wen, Y. Liang, X. Liu, J. Zhao, X. Long, Irreversible electroporation-induced inflammation facilitates neutrophil-mediated drug delivery to enhance pancreatic cancer therapy. Mol. Pharm. 21, 1998–2011 (2024). 38412284 10.1021/acs.molpharmaceut.4c00006

[34] A. Mantovani, P. Allavena, F. Marchesi, C. Garlanda, Macrophages as tools and targets in cancer therapy. Nat. Rev. Drug Discov. 21, 799–820 (2022). 35974096 10.1038/s41573-022-00520-5 PMC9380983

[35] S. Jin, C. F. Guerrero-Juarez, L. Zhang, I. Chang, R. Ramos, C. H. Kuan, P. Myung, M. V. Plikus, Q. Nie, Inference and analysis of cell-cell communication using CellChat. Nat. Commun. 12, 1088 (2021). 33597522 10.1038/s41467-021-21246-9 PMC7889871

[36] S. Cheng, Z. Li, R. Gao, B. Xing, Y. Gao, Y. Yang, S. Qin, L. Zhang, H. Ouyang, P. Du, L. Jiang, B. Zhang, Y. Yang, X. Wang, X. Ren, J.-X. Bei, X. Hu, Z. Bu, J. Ji, Z. Zhang, A pan-cancer single-cell transcriptional atlas of tumor infiltrating myeloid cells. Cell 184, 792–809.e23 (2021). 33545035 10.1016/j.cell.2021.01.010

[37] N. V. Serbina, E. G. Pamer, Monocyte emigration from bone marrow during bacterial infection requires signals mediated by chemokine receptor CCR2. Nat. Immunol. 7, 311–317 (2006). 16462739 10.1038/ni1309

[38] B.-Z. Qian, J. Li, H. Zhang, T. Kitamura, J. Zhang, L. R. Campion, E. A. Kaiser, L. A. Snyder, J. W. Pollard, CCL2 recruits inflammatory monocytes to facilitate breast-tumour metastasis. Nature 475, 222–225 (2011). 21654748 10.1038/nature10138 PMC3208506

[39] H. Raghu, C. M. Lepus, Q. Wang, H. H. Wong, N. Lingampalli, F. Oliviero, L. Punzi, N. J. Giori, S. B. Goodman, C. R. Chu, J. B. Sokolove, W. H. Robinson, CCL2/CCR2, but not CCL5/CCR5, mediates monocyte recruitment, inflammation and cartilage destruction in osteoarthritis. Ann. Rheum. Dis. 76, 914–922 (2017). 27965260 10.1136/annrheumdis-2016-210426 PMC5834918

[40] C. Cui, C. Xu, W. Yang, Z. Chi, X. Sheng, L. Si, Y. Xie, J. Yu, S. Wang, R. Yu, J. Guo, Y. Kong, Ratio of the interferon-γ signature to the immunosuppression signature predicts anti-PD-1 therapy response in melanoma. npj Genom. Med. 6, 7 (2021). 33542239 10.1038/s41525-021-00169-w PMC7862369

[41] S. P. Arlauckas, S. B. Garren, C. S. Garris, R. H. Kohler, J. Oh, M. J. Pittet, R. Weissleder, Arg1 expression defines immunosuppressive subsets of tumor-associated macrophages. Theranostics 8, 5842–5854 (2018). 30613266 10.7150/thno.26888 PMC6299430

[42] Y. Tie, F. Tang, Y. Q. Wei, X. W. Wei, Immunosuppressive cells in cancer: Mechanisms and potential therapeutic targets. J. Hematol. Oncol. 15, 61 (2022). 35585567 10.1186/s13045-022-01282-8 PMC9118588

[43] T. M. Nywening, A. Wang-Gillam, D. E. Sanford, B. A. Belt, R. Z. Panni, B. M. Cusworth, A. T. Toriola, R. K. Nieman, L. A. Worley, M. Yano, K. J. Fowler, A. C. Lockhart, R. Suresh, B. R. Tan, K.-H. Lim, R. C. Fields, S. M. Strasberg, W. G. Hawkins, D. G. DeNardo, S. P. Goedegebuure, D. C. Linehan, Targeting tumour-associated macrophages with CCR2 inhibition in combination with FOLFIRINOX in patients with borderline resectable and locally advanced pancreatic cancer: A single-centre, open-label, dose-finding, non-randomised, phase 1b trial. Lancet Oncol. 17, 651–662 (2016). 27055731 10.1016/S1470-2045(16)00078-4 PMC5407285

[44] M. Cannistrà, M. Ruggiero, A. Zullo, S. Serafini, R. Grande, B. Nardo, Metastases of pancreatic adenocarcinoma: A systematic review of literature and a new functional concept. Int. J. Surg. 21, S15–S21 (2015). 26123383 10.1016/j.ijsu.2015.04.093

[45] M. Enamorado, S. Iborra, E. Priego, F. J. Cueto, J. A. Quintana, S. Martinez-Cano, E. Mejias-Perez, M. Esteban, I. Melero, A. Hidalgo, D. Sancho, Enhanced anti-tumour immunity requires the interplay between resident and circulating memory CD8^+^ T cells. Nat. Commun. 8, 16073 (2017). 28714465 10.1038/ncomms16073 PMC5520051

[46] Q. Liu, Z. Sun, L. Chen, Memory T cells: Strategies for optimizing tumor immunotherapy. Protein Cell 11, 549–564 (2020). 32221812 10.1007/s13238-020-00707-9 PMC7381543

[47] B. Rubinsky, G. Onik, P. Mikus, Irreversible electroporation: A new ablation modality — clinical implications. Technol. Cancer Res. Treat. 6, 37–48 (2007). 17241099 10.1177/153303460700600106

[48] E. W. Lee, C. Chen, V. E. Prieto, S. M. Dry, C. T. Loh, S. T. Kee, Advanced hepatic ablation technique for creating complete cell death: Irreversible electroporation. Radiology 255, 426–433 (2010). 20413755 10.1148/radiol.10090337

[49] N. Zhang, Z. Li, X. Han, Z. Zhu, Z. Li, Y. Zhao, Z. Liu, Y. Lv, Irreversible electroporation: An emerging immunomodulatory therapy on solid tumors. Front. Immunol. 12, 811726 (2022). 35069599 10.3389/fimmu.2021.811726 PMC8777104

