# Supplementary material for: Targeting tumor-associated CCR2+ macrophages to inhibit pancreatic cancer recurrence following irreversible electroporation
Source: Sci Adv. 2025 Jul 23;11(30):eadw2937. doi: 10.1126/sciadv.adw2937 (PMC12285706; doi:10.1126/sciadv.adw2937)
Supplement: Supplementary file 1 — Figs. S1 to S23 [file sciadv.adw2937_sm.pdf]

Science Advances
AAAS

Supplementary Materials for

Targeting tumor-associated CCR2$^+$ macrophages to inhibit pancreatic cancer recurrence following irreversible electroporation

Weichen Xu *et al.*

Corresponding author: Wenwen Yue, yuewen0902@163.com; Liping Sun, sunliping_s@126.com; Huixiong Xu, xu.huixiong@zs-hospital.sh.cn

Sci. Adv. **11**, eadw2937 (2025)
DOI: 10.1126/sciadv.adw2937

This PDF file includes:

Figs. S1 to S23

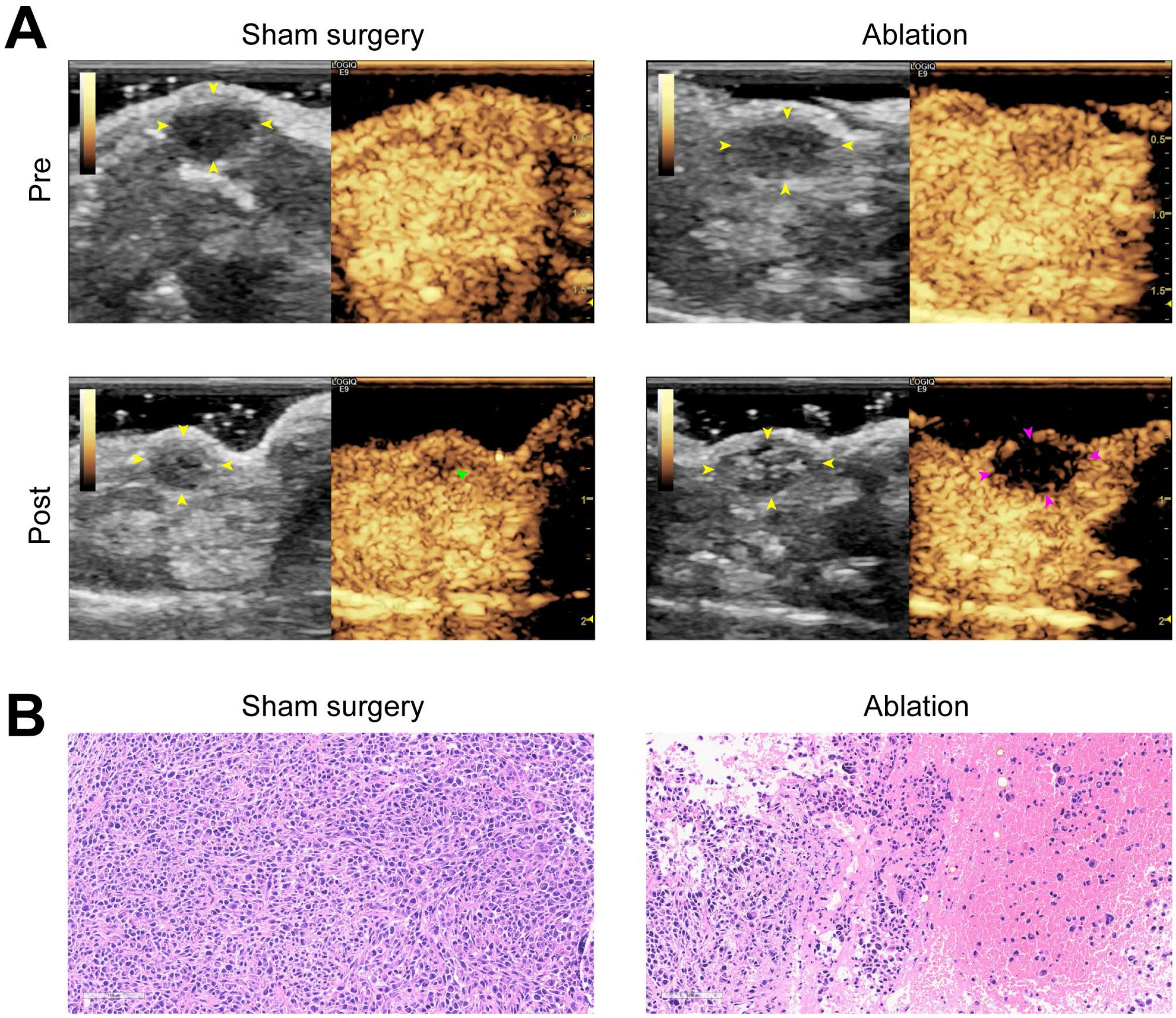

Fig. S1.

Evaluation of ablation efficacy. (**A**) Contrast-enhanced ultrasound (CEUS) images of subcutaneous KPC tumor pre- and post-ablation. Yellow arrows point to the tumor margins on grayscale ultrasound images, green arrows point to the puncture tract within the tumor in the sham group, and pink arrows point to the non-enhanced areas on the CEUS images. (**B**) Representative staining image of H&E (Scale bar = 100 μm).

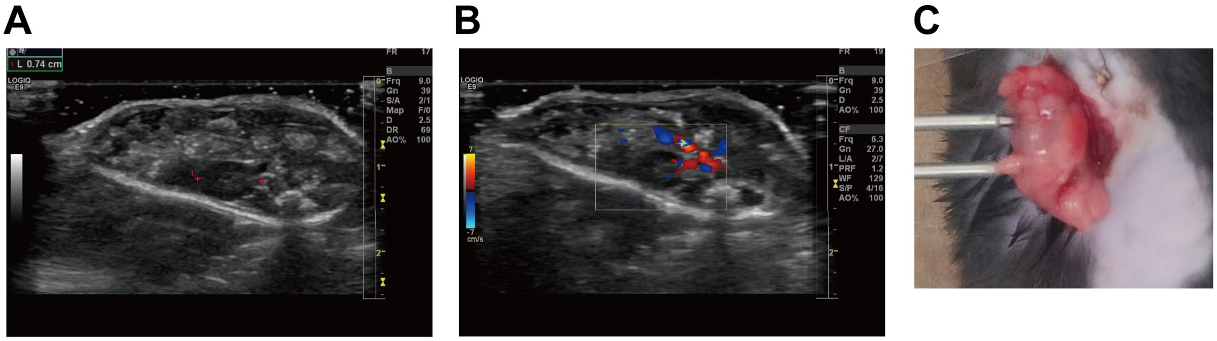

Fig. S2.

The establishment of orthotopic KPC tumor ablation model. (**A**) Representative grayscale ultrasound image showing a hypoechoic mass in mouse pancreas (d = 0.74 cm). (**B**) Representative Doppler ultrasound image of blood flow signals around the mass. (**C**) Representative image of IRE treatment for the orthotopic pancreatic tumor in mouse.

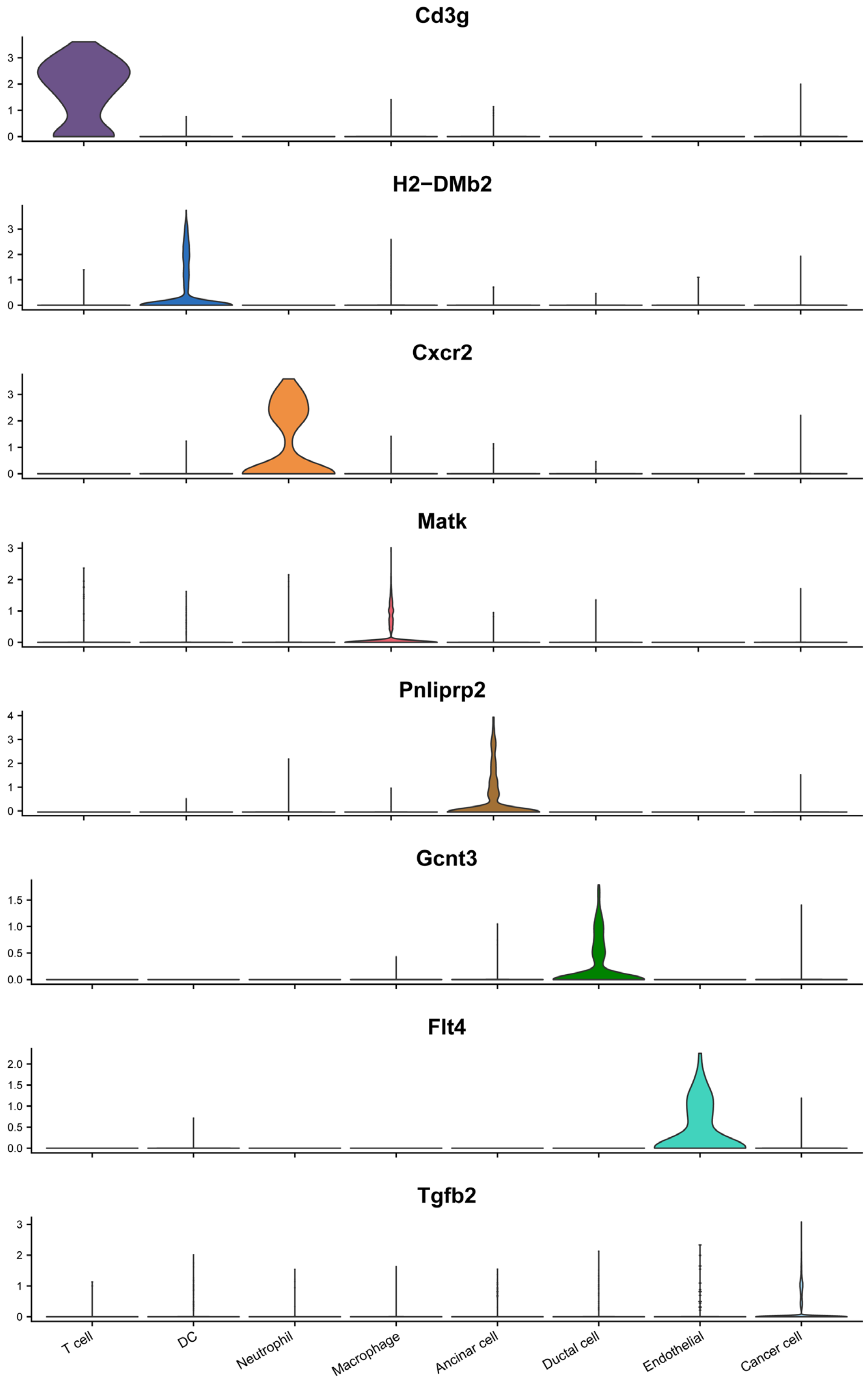

Fig. S3.

Cell type identification in PDAC tumor by single-cell sequencing. Violin plots showing the expression levels of representative markers for each cell type.

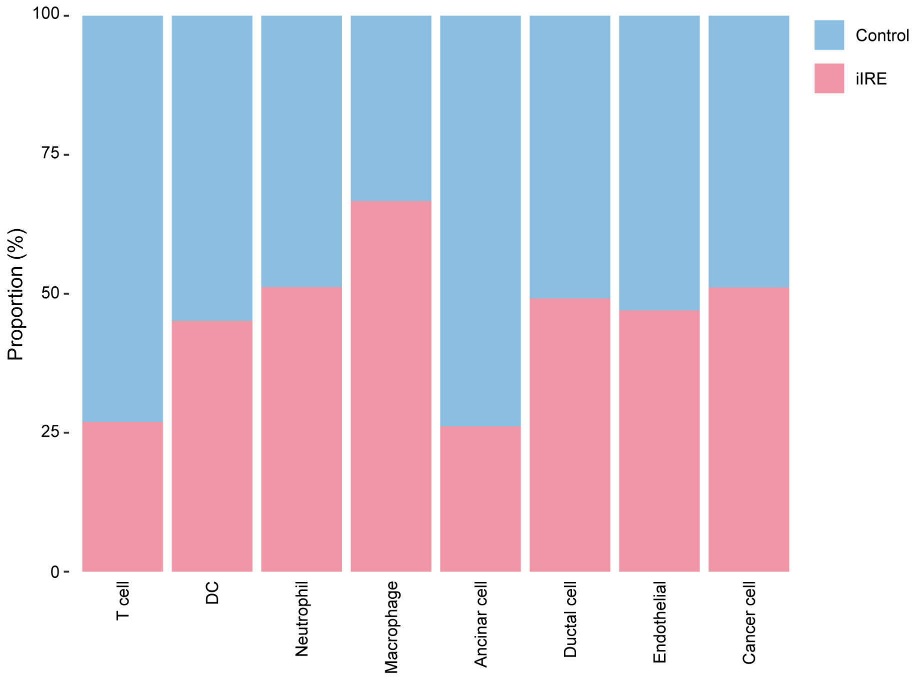

Fig. S4.

Cell type proportion of the control and iIRE group in single-cell RNA sequencing analysis.

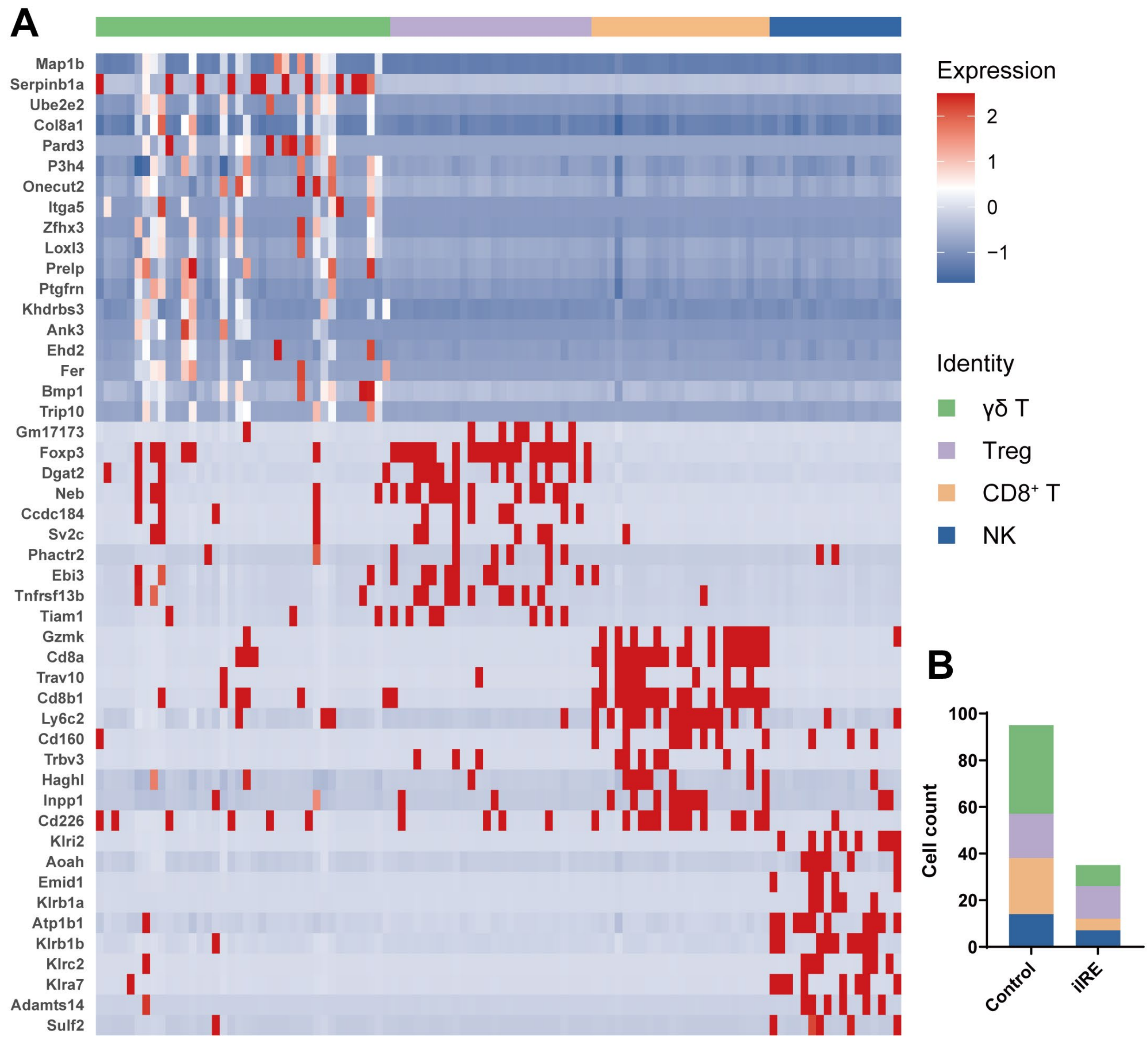

Fig. S5.

Identification of T cell subtypes. (**A**) Heatmap displaying the top 10 markers for T cell subtypes: Cluster 1: γδ T cell; Cluster 2: Treg cell; Cluster 3: $CD8^+$ T cell; Cluster 4: NK cell. (**B**) Numbers of T cells in the control and iIRE groups.

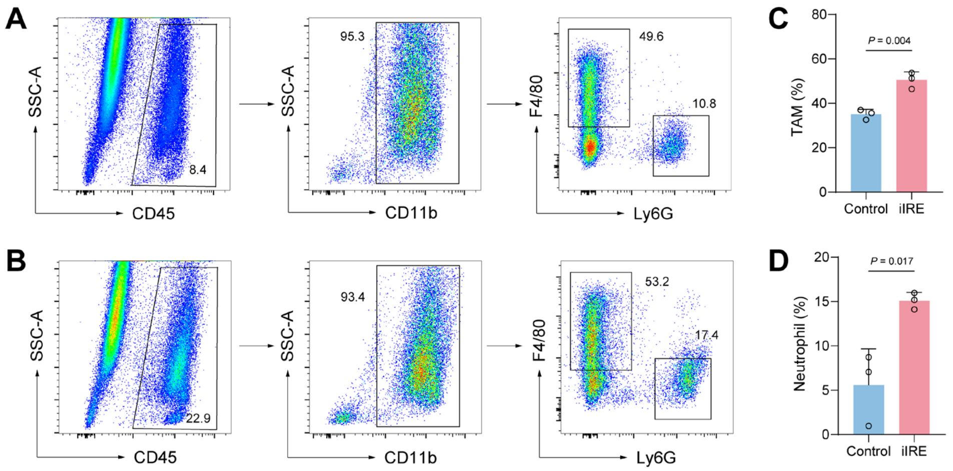

Fig. S6.

Proportion of tumor-associated macrophages (TAMs) and neutrophils in the control and iIRE KPC tumors. (**A, B**) Representative flow cytometry analysis of TAM (Ly6G$^-$F4/80$^+$CD11b$^+$CD45$^+$) and neutrophil (Ly6G$^+$F4/80$^-$CD11b$^+$CD45$^+$) in the control tumors (A) and iIRE tumors (B). (**C**) Quantitative analysis of TAM (n = 3). (**D**) Quantitative analysis of neutrophil (n = 3). Data are expressed as mean ± SD. Statistical differences were calculated using Student's *t* test.

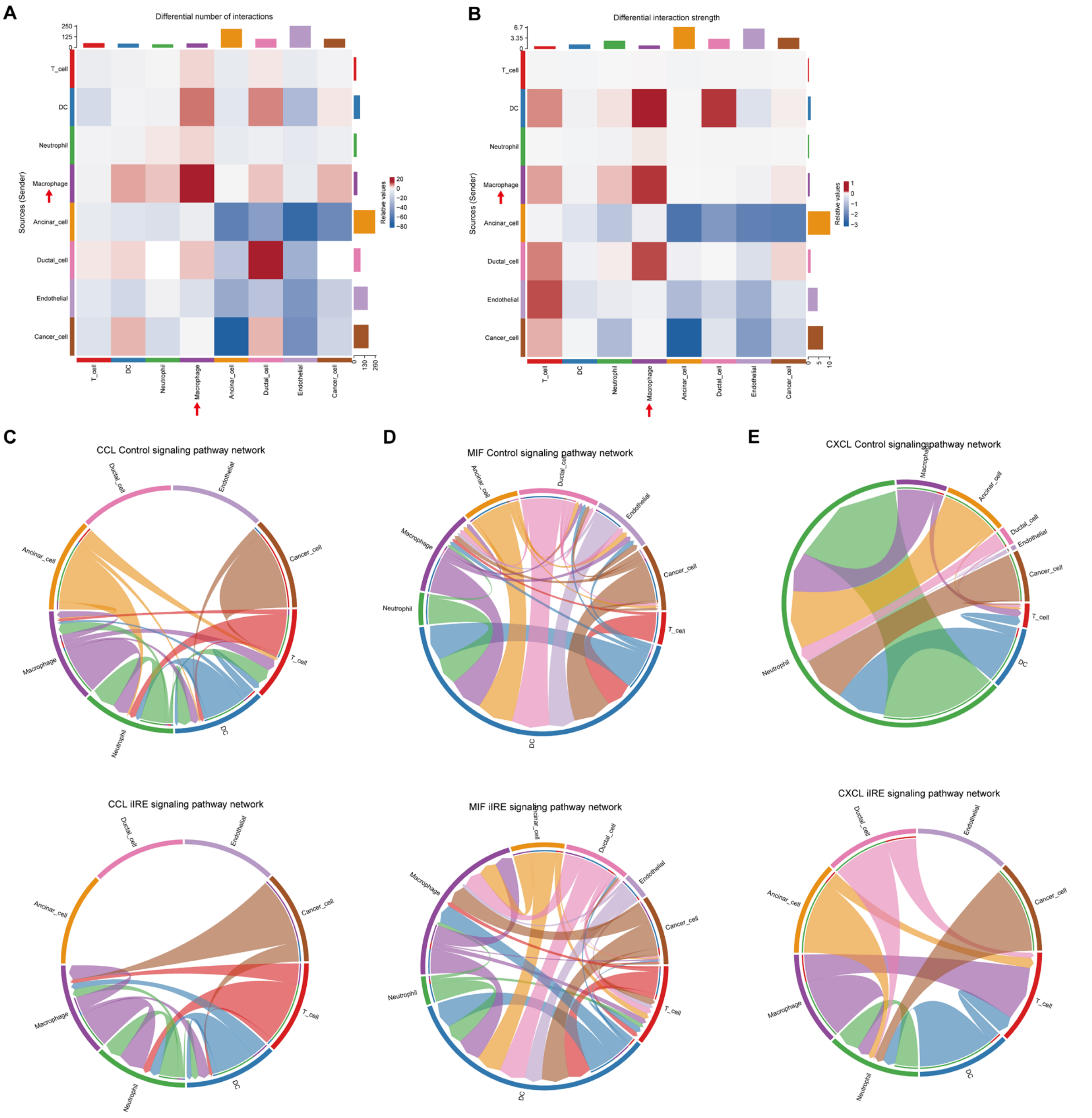

Fig. S7.

CellChat analysis. (**A**) Heatmap comparing the number of cell-cell interactions between the iIRE group and the Control group, with red and blue representing increased and decreased interaction numbers, respectively. The vertical axis indicates ligand cell types, while the horizontal axis indicates receptor cell types. (**B**) Heatmap comparing the strength of cell-cell interactions between the iIRE group and the Control group, with red and blue representing increased and decreased interaction strengths, respectively. (**C–E**) Chord diagram illustrating bidirectional cell communication via (C) CCL signaling pathways, (D) MIF signaling pathways and (E) CXCL signaling pathways. The outer ring represents ligand cell types, while the inner ring corresponds to the receptor cell types.

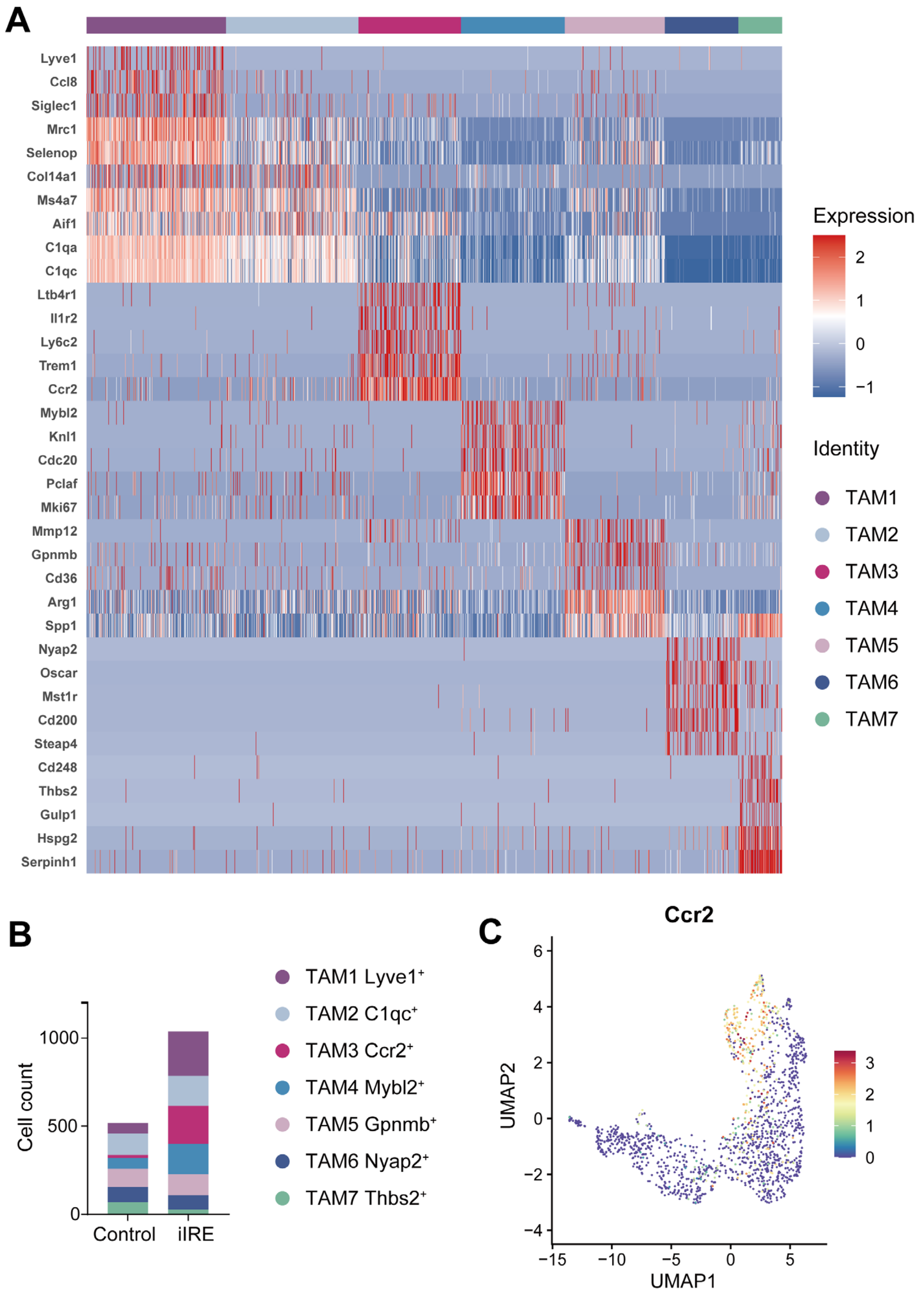

Fig. S8.

Identification of macrophage subtypes. (**A**) Heatmap displaying the top 5 markers for each macrophage subtype. (**B**) Cell count of the 7 TAM subclusters. (**C**) UMAP plot showing the expression levels of the CCR2 gene in macrophages. Abbreviation: UMAP, uniform manifold approximation and projection.

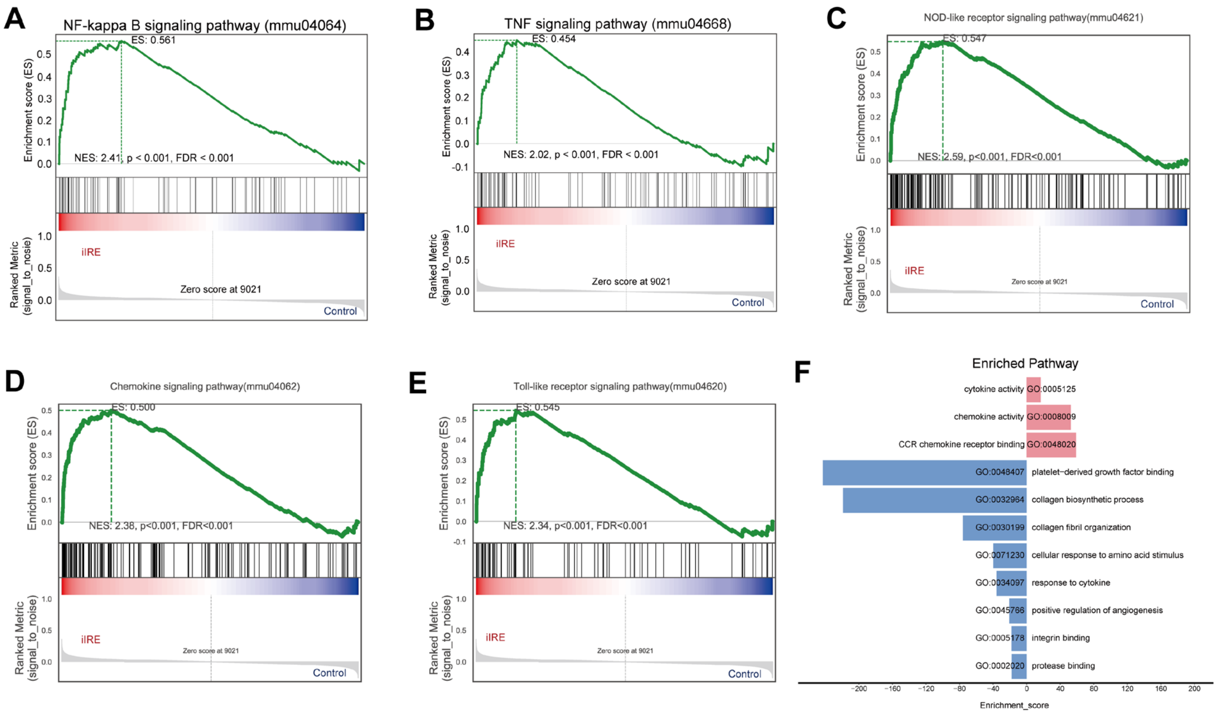

Fig. S9.

Enrichment analysis of DEGs in macrophage. (**A–E**) GSEA of KEGG pathway including NF-kappa B signaling pathway, TNF signaling pathway, NOD-like receptor signaling pathway, chemokine signaling pathway and Toll-like receptor signaling pathway. (**F**) Bar plot displaying enrichment score of GO biological process. Abbreviations: DEG, differential expressed gene; GSEA, gene set enrichment analysis; GO, gene ontology.

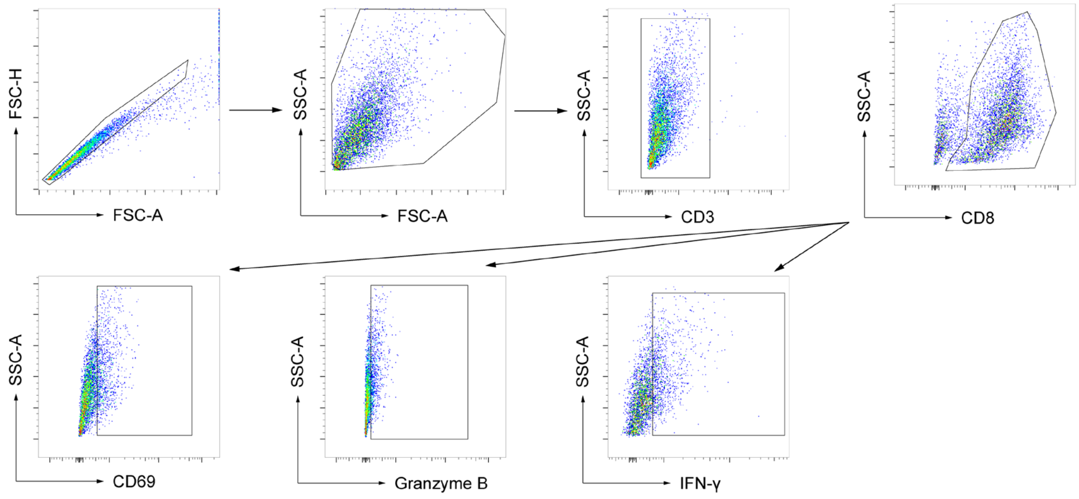

Fig. S10.

Flow cytometry gating strategy of co-cultured cells. Gating strategy for cytotoxic T cells: $CD69^+$ ($CD69^+CD8^+CD3^+$), Granzyme B^+ (Granzyme $B^+CD8^+CD3^+$), and IFN-γ^+ (IFN-γ^+ $CD8^+CD3^+$) $CD8^+$ T cells.

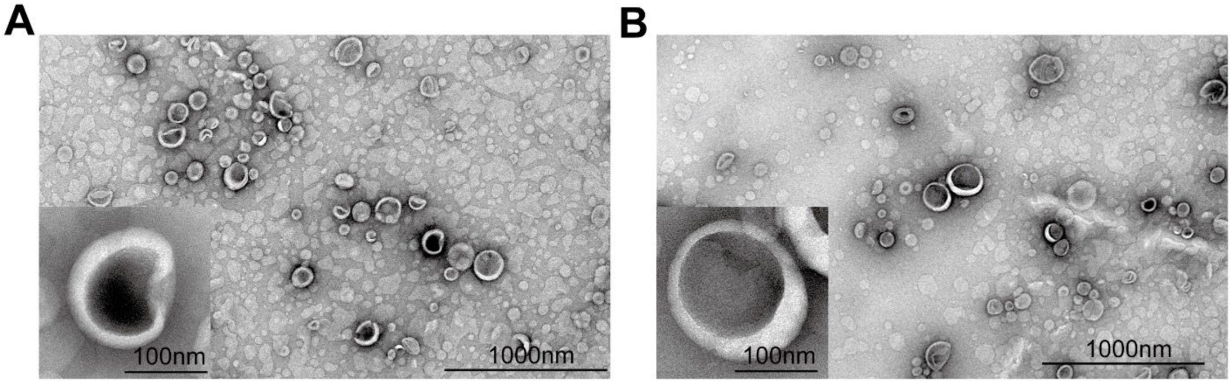

Fig. S11.

TEM images. (**A**) PF/GEM@Liposomes. (**B**)PF/GEM@mPLVs.

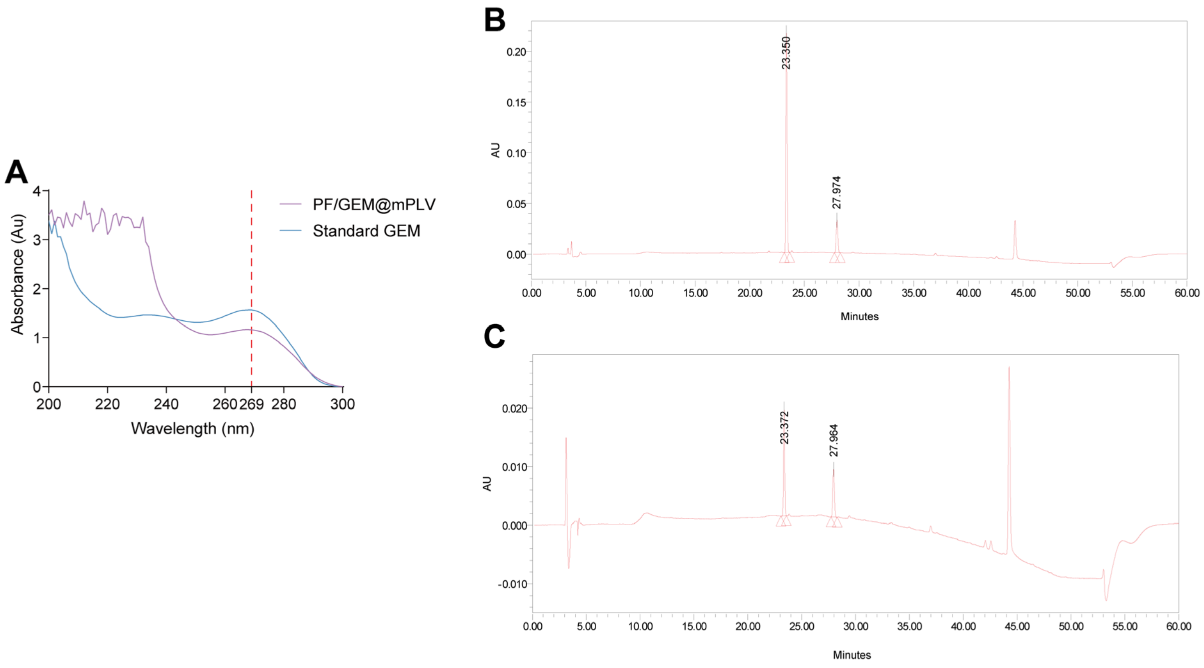

Fig. S12.

UV-vis and HPLC spectra. (**A**) Representative UV-vis spectra for gemcitabine standard and PF/GEM@mPLV. (**B, C**) Representative HPLC chromatograms for (B) PF standard and (C) PF/GEM@mPLV.

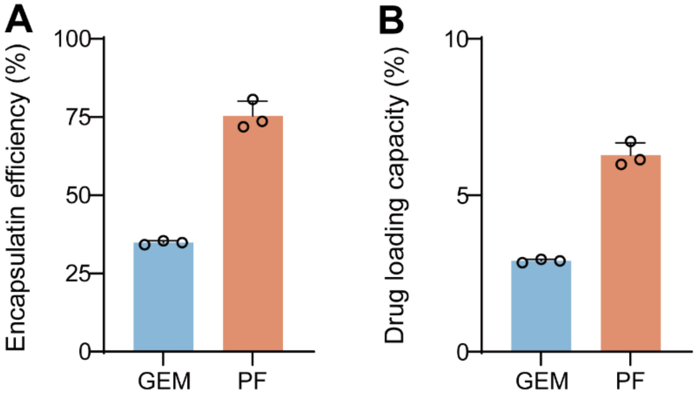

Fig. S13.

(A) Encapsulation efficiency and (B) loading capacity of PF/GEM@mPLV for gemcitabine (GEM) and PF-4136309 (PF). Data are expressed as mean ± SD (n = 3). Statistical differences were calculated using Student's *t* test.

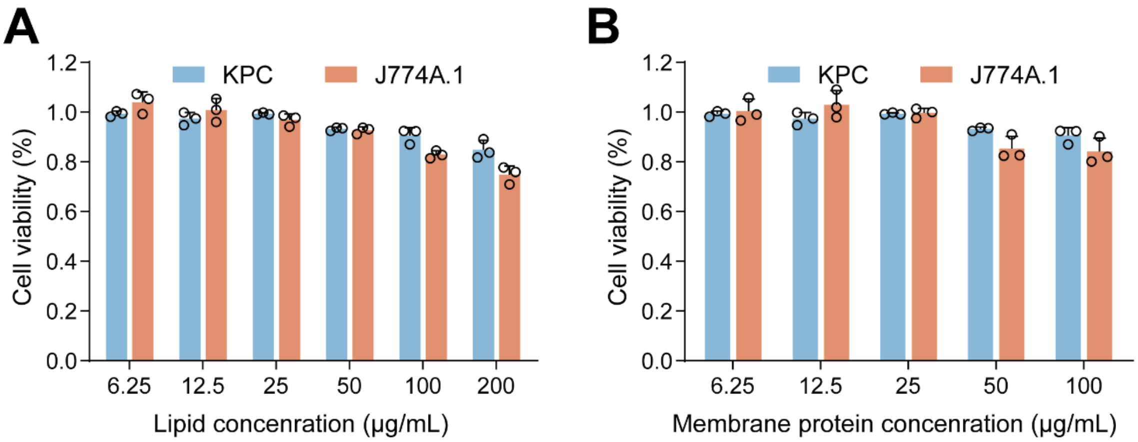

Fig. S14.

In-vitro biocompatibility of liposome and mPLV. Bar plots displaying cell viability of KPC cells and J774A.1 cells treated with varying concentrations of (**A**) lipid and (**B**) macrophage membrane protein.

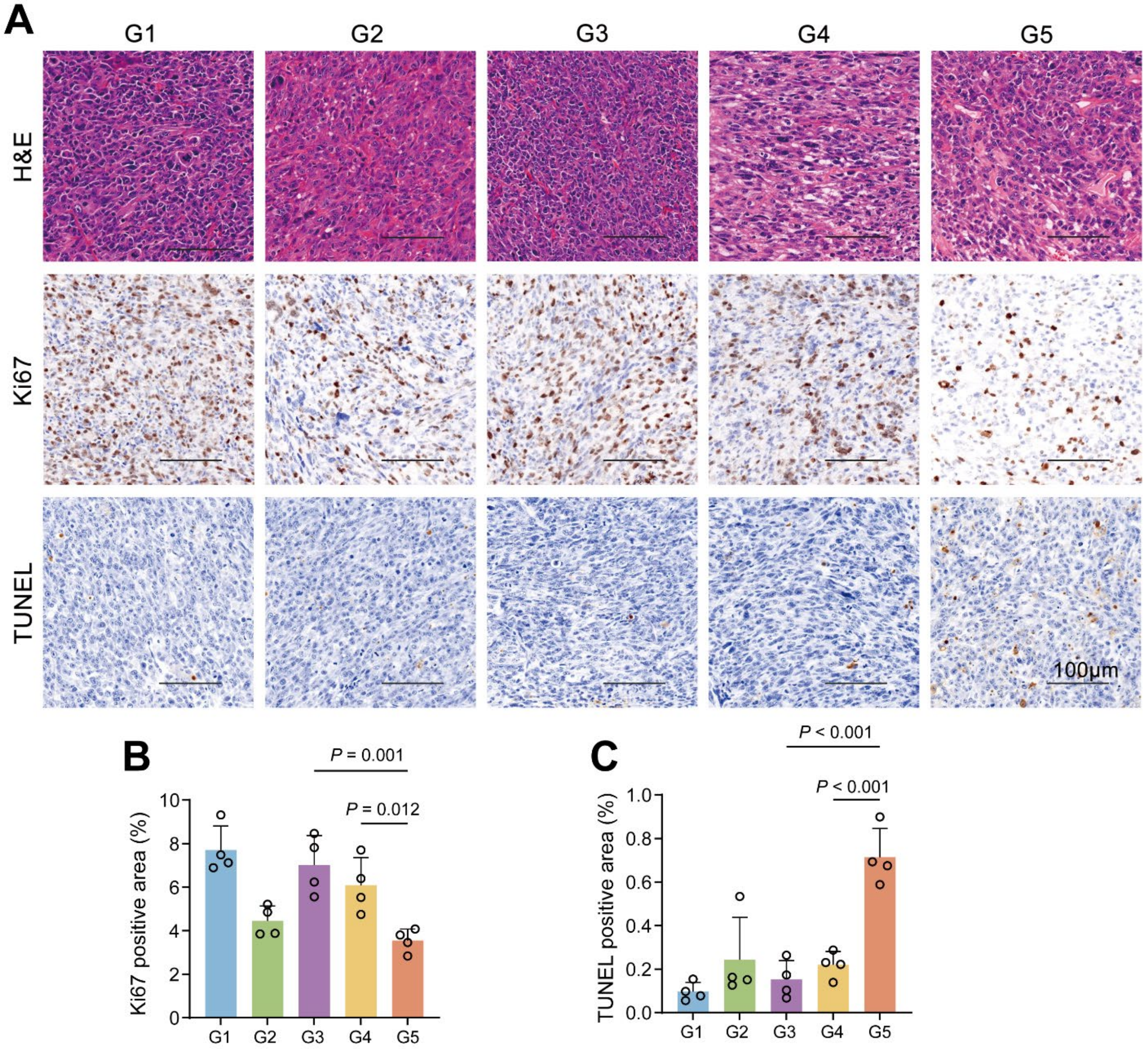

Fig. S15.

The viability and necrosis status of tumor tissues after various treatments, as shown by H&E staining, Ki67 assay and TUNEL assay. (**A**) Representative staining image of tumor tissues (Scale bar = 100 μm). (**B**) Quantification of Ki67 positive area (n = 4). (**C**) Quantification of TUNEL positive area (n = 4). Data are expressed as mean ± SD. Statistical differences were calculated using one-way ANOVA.

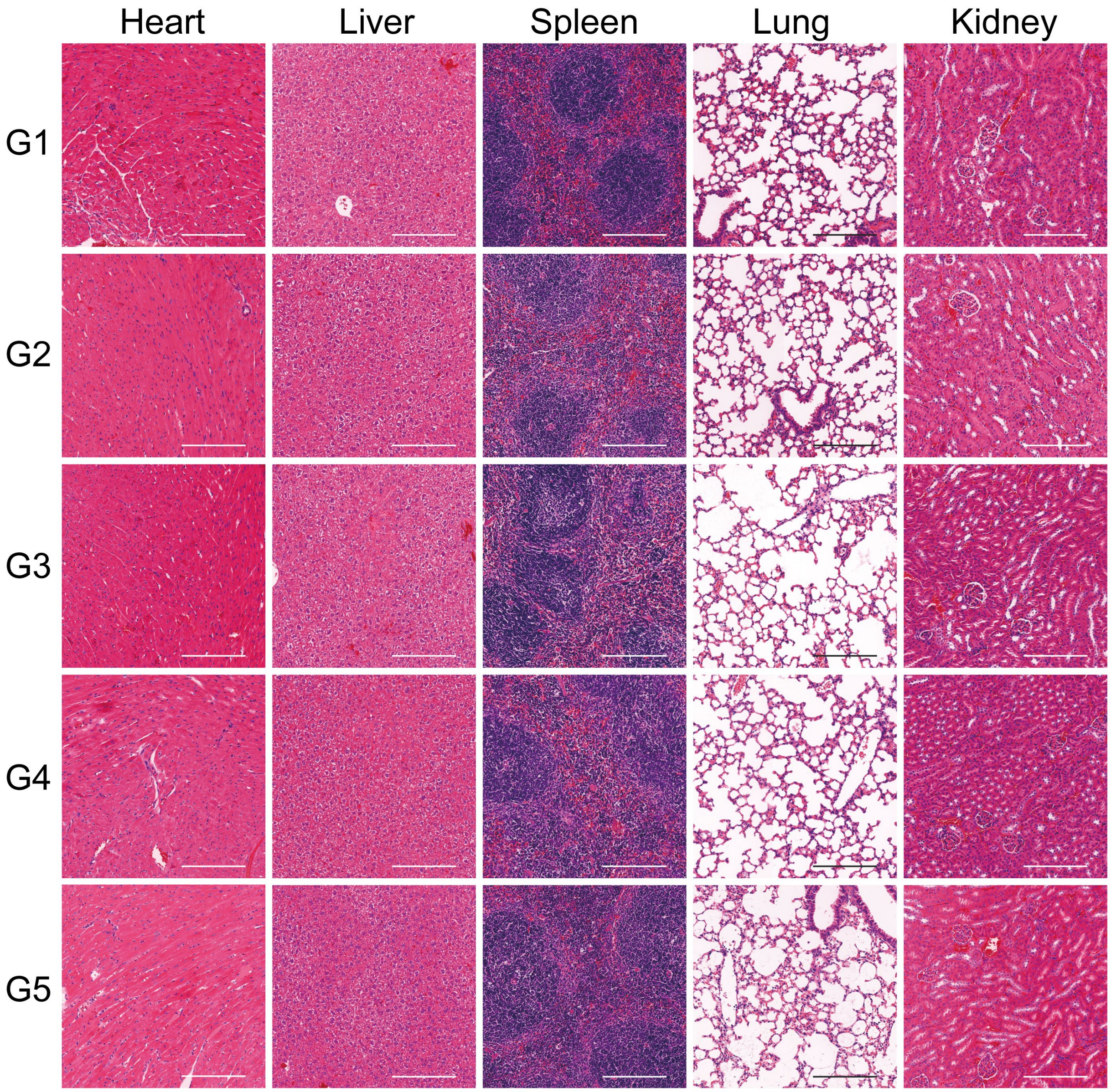

Fig. S16.

Representative H&E staining of major organs in subcutaneous tumor mouse model after intravenous administration of PBS (G1), free PF + GEM (G2), PF@mPLV (G3), GEM@mPLV (G4), and PF/GEM@mPLV (G5) (Scale bar = 200 μm).

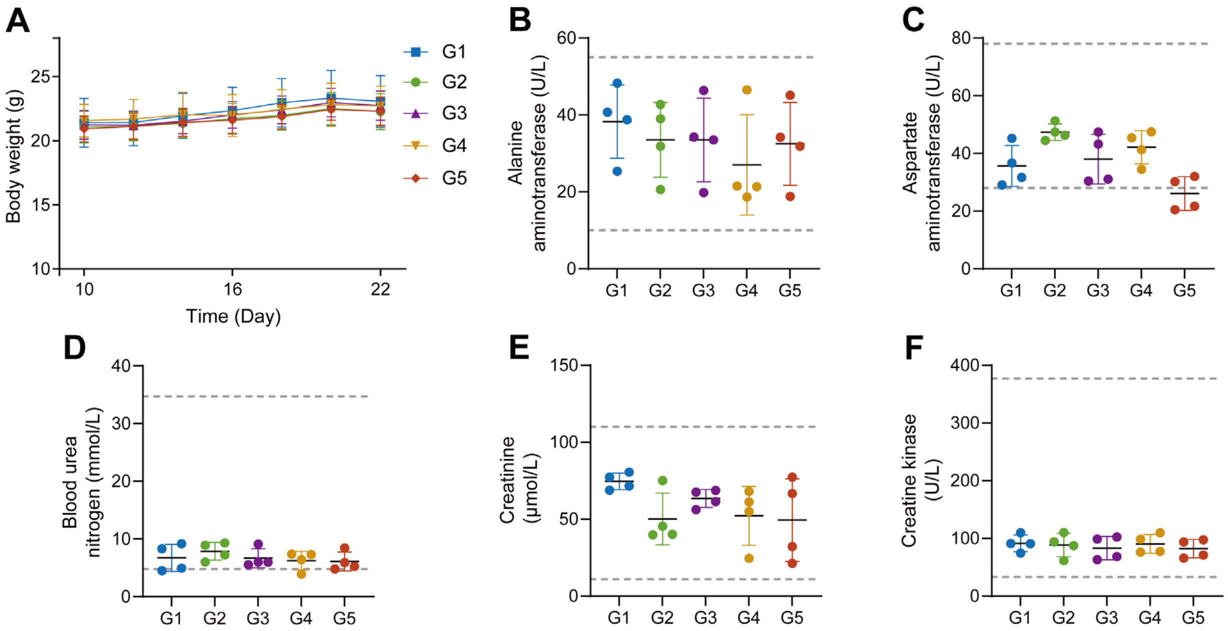

Fig. S17.

Biosafety evaluation in subcutaneous tumor mouse model. (**A**) Body weight of mice treated with PBS (G1), free PF + GEM (G2), PF@mPLV (G3), GEM@mPLV (G4), and PF/GEM@mPLV (G5) (n = 10). (**B**) Serum levels of alanine aminotransferase (ALT) (n = 4). (**C**) Serum levels of aspartate aminotransferase (AST) (n = 4). (**D**) Serum levels of blood urea nitrogen (BUN) (n = 4). (**E**) Serum levels of creatinine (n = 4). (**F**) Serum levels of Creatine kinase (CK) (n = 4). The normal range of blood chemistry parameters is indicated by gray dashed lines. Data are expressed as mean ± SD. Statistical differences were calculated using one-way ANOVA.

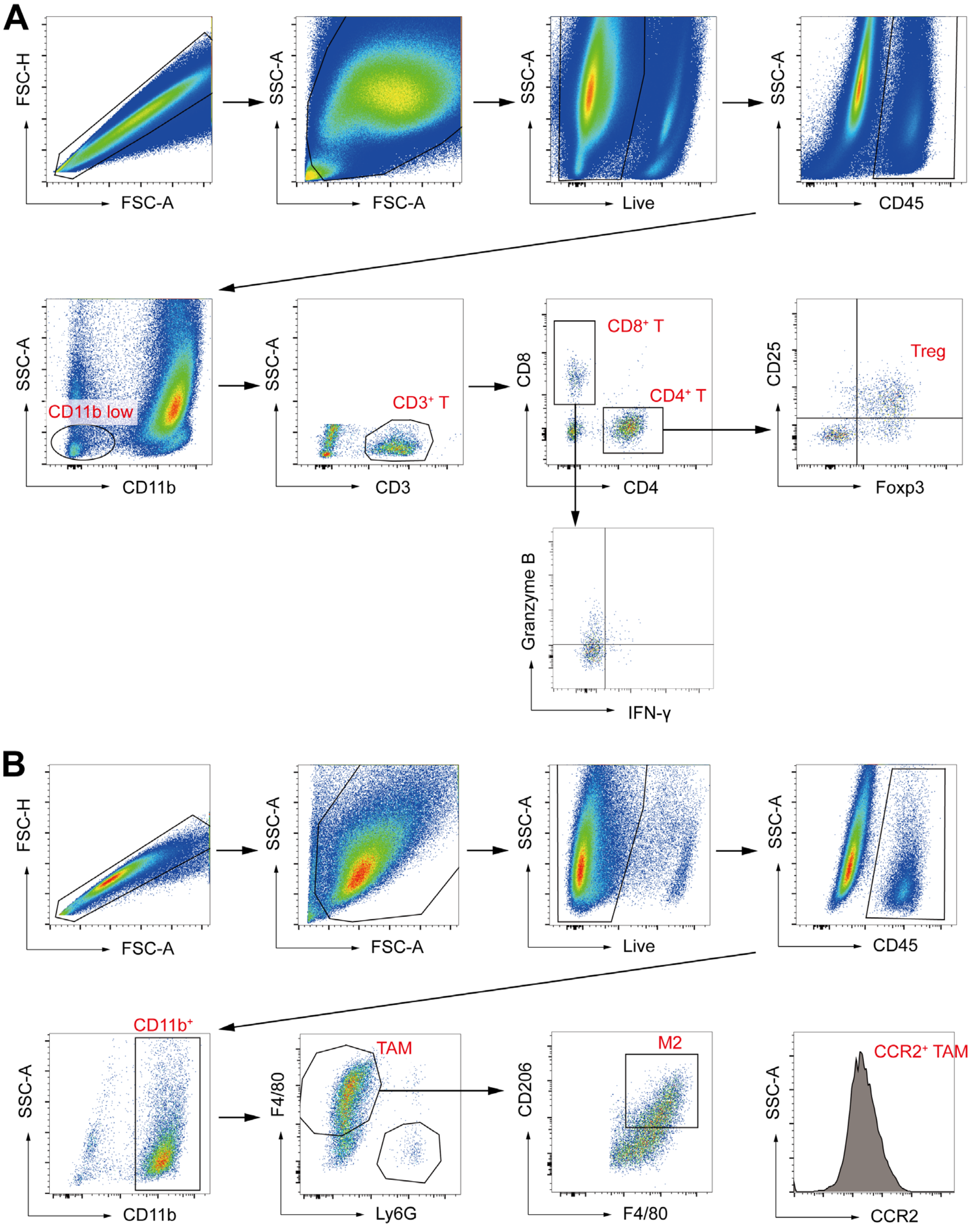

Fig. S18.

Flow cytometry gating strategy of subcutaneous tumor. (**A**) Gating strategy for $CD4^+$ T cell ($CD4^+CD3^+CD45^+$), $CD8^+$ T cell ($CD8^+CD3^+CD45^+$), Granzyme B^+CD8^+ T cell, IFN-γ^+CD8^+ T cell and Treg cell ($CD25^+Foxp3^+CD4^+CD3^+CD45^+$). (**B**) Gating strategy for TAM ($Ly6G^-$ $F4/80^+CD11b^+CD45^+$), M2-TAM ($CD206^{hi}Ly6G^-F4/80^+CD11b^+CD45^+$) and $CCR2^+$ TAM ($CCR2^+Ly6G^-F4/80^+CD11b^+CD45^+$).

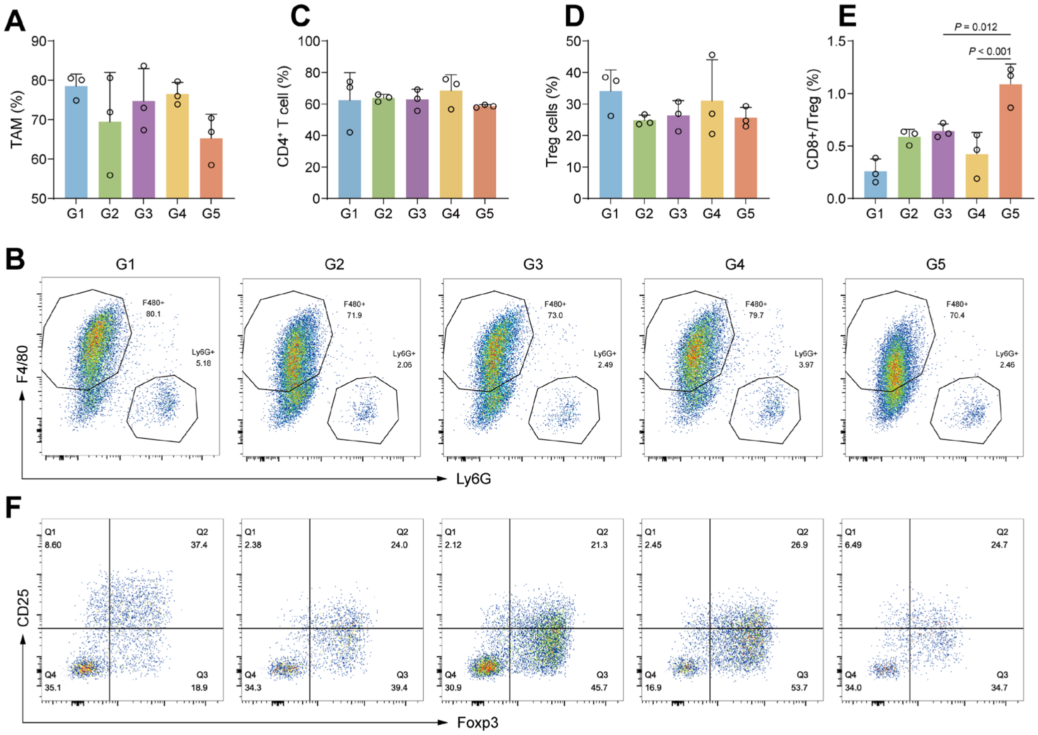

Fig. S19.

Immune response in the subcutaneous tumor model. (**A**) Quantitative analysis of TAM (n = 3). (**B**) Representative flow cytometry analysis of TAM (Ly6G$^-$F4/80$^+$CD11b$^+$CD45$^+$). (**C**) Quantitative analysis of CD4$^+$ T cell (CD4$^+$CD3$^+$CD45$^+$) (n = 3). (**D**) Quantitative analysis of Treg cell (n = 3). (**E**) Quantitative analysis of CD8$^+$/Treg ratio (n = 3). (**F**) Representative flow cytometry analysis of Treg cell (CD25$^+$Foxp3$^+$CD4$^+$CD3$^+$CD45$^+$). Data are expressed as mean ± SD. Statistical differences were calculated using one-way ANOVA.

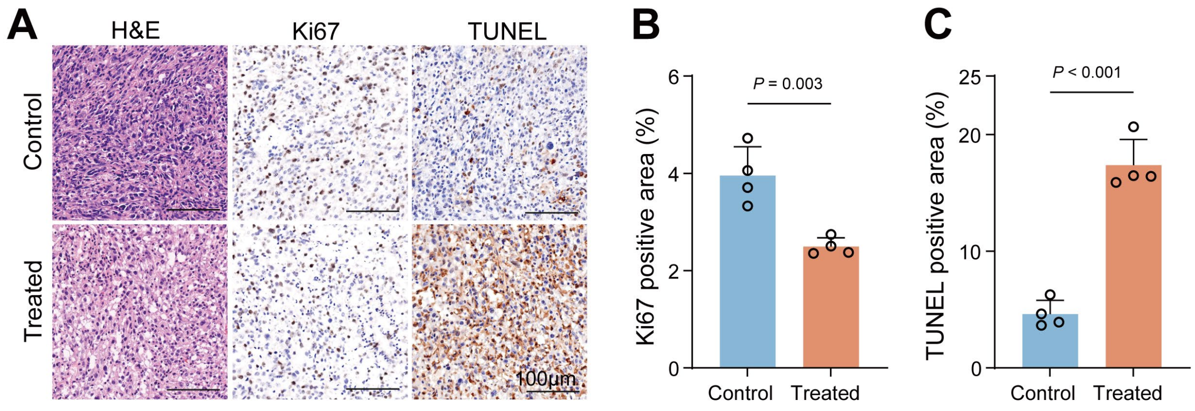

Fig. S20.

The viability and necrosis status of tumor tissues in the orthotopic tumor model after various treatments, shown by H&E staining, Ki67 assay and TUNEL assay. (**A**) Representative images of H&E, Ki67, and TUNEL staining of tumor tissues (Scale bar = 100 μm). (**B, C**) Quantitative analysis of Ki67-positive (B) area and TUNEL-positive (C) area ($n = 4$). Data are expressed as mean ± SD. Statistical differences were calculated using Student's *t* test.

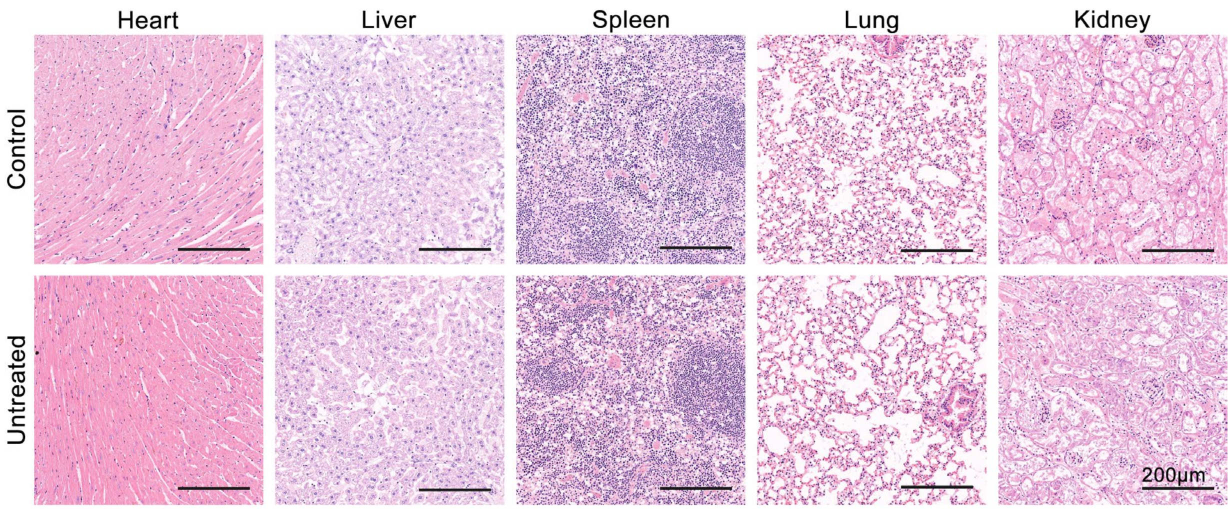

Fig. S21.

Representative H&E staining images of major organs from orthotopic KPC tumor-bearing mice (Scale bar = 200 μm).

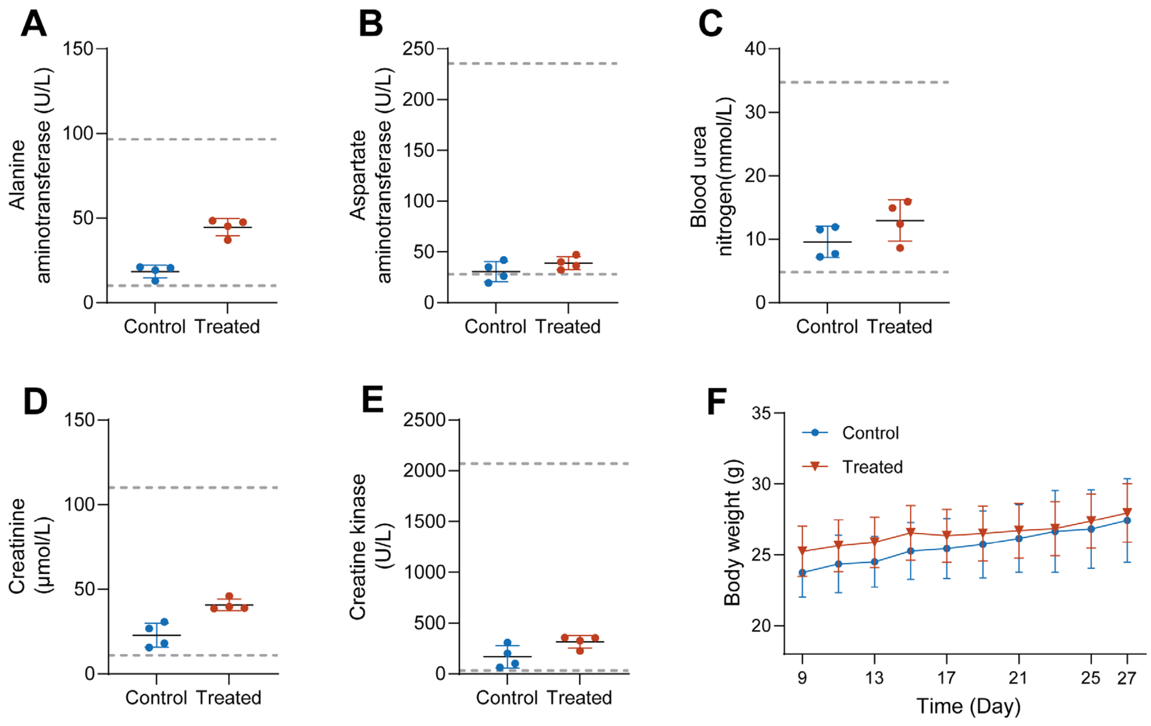

Fig. S22.

Blood chemistry analyses and body weight of PF/GEM@mPLV treatment in orthotopic KPC tumor mouse model. (**A**) Serum levels of alanine aminotransferase (ALT) (n = 4). (**B**) Serum levels of aspartate aminotransferase (AST) (n = 4). (**C**) Serum levels of blood urea nitrogen (BUN) levels (n = 4). (**D**) Serum levels of creatinine (n = 4). (**E**) Serum levels of Creatine kinase (CK) (n = 4). (**F**) Body weight (n = 10). Data are expressed as mean ± SD. Statistical differences were calculated using Student's *t* test.

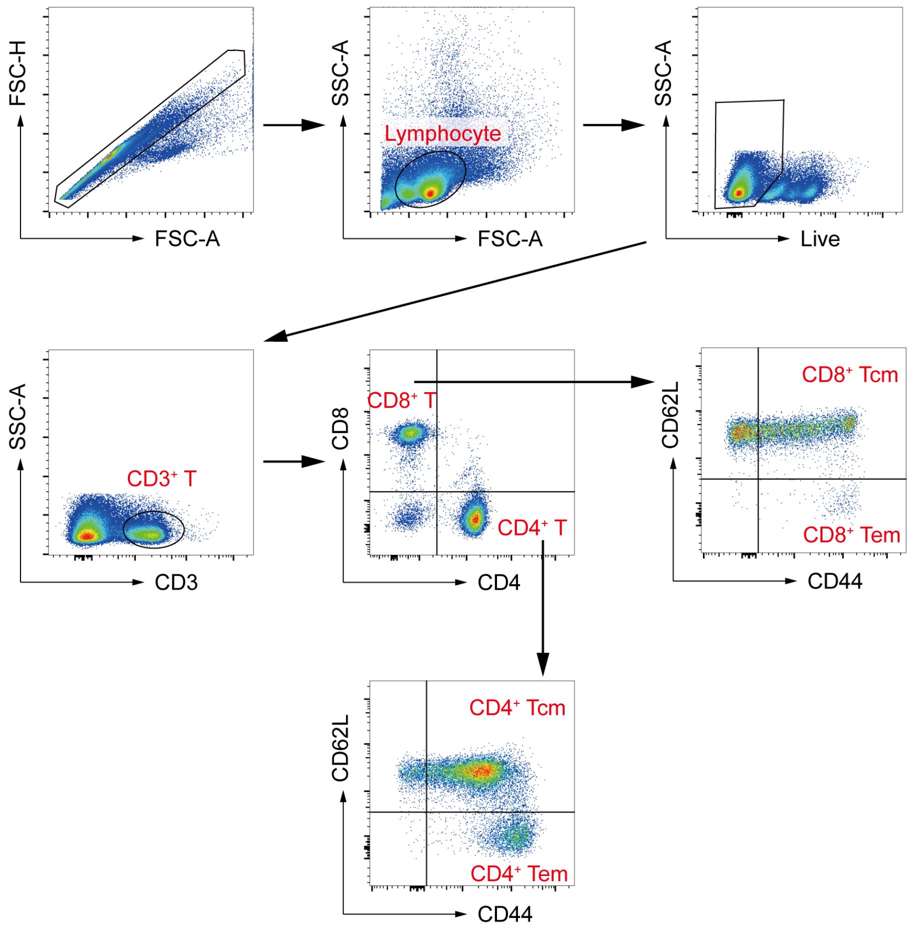

Fig. S23.

Flow cytometry gating strategy for memory T cells in the spleen of the orthotopic tumor model. Central memory T cell (Tcm, CD62L$^+$CD44$^+$) and effector memory T cell (Tem, CD62L$^-$CD44$^+$) subsets from CD8$^+$ and CD4$^+$ T cells are shown separately.
